# Supramolecular Self-Assembly of Porphyrin and Metallosurfactant as a Drug Nanocontainer Design

**DOI:** 10.3390/nano12121986

**Published:** 2022-06-09

**Authors:** Ruslan R. Kashapov, Yuliya S. Razuvayeva, Svetlana S. Lukashenko, Syumbelya K. Amerhanova, Anna P. Lyubina, Alexandra D. Voloshina, Victor V. Syakaev, Vadim V. Salnikov, Lucia Y. Zakharova

**Affiliations:** 1 Arbuzov Institute of Organic and Physical Chemistry, FRC Kazan Scientific Center of RAS, 8 Arbuzov Street, 420088 Kazan, Russia; razuvayeva.yuliya@iopc.ru (Y.S.R.); lukashenko@iopc.ru (S.S.L.); syumbelya07@mail.ru (S.K.A.); aplyubina@gmail.com (A.P.L.); microbi@iopc.ru (A.D.V.); vsyakaev@iopc.ru (V.V.S.); lucia@iopc.ru (L.Y.Z.); 2 Kazan Institute of Biochemistry and Biophysics, FRC Kazan Scientific Center of RAS, 2/31 Lobachevsky Street, 420111 Kazan, Russia; salnikov_russ@yahoo.com

**Keywords:** porphyrins, amphiphiles, cisplatin, self-assembly, nanostructures

## Abstract

The combined method of treating malignant neoplasms using photodynamic therapy and chemotherapy is undoubtedly a promising and highly effective treatment method. The development and establishment of photodynamic cancer therapy is closely related to the creation of sensitizers based on porphyrins. The present study is devoted to the investigation of the spectroscopic, aggregation, and solubilization properties of the supramolecular system based on 5,10,15,20-tetrakis(4-sulfonatophenyl)porphyrin (TSPP) and lanthanum-containing surfactant (LaSurf) in an aqueous medium. The latter is a complex of lanthanum nitrate and two cationic amphiphilic molecules of 4-aza-1-hexadecylazoniabicyclo[2.2.2]octane bromide. The mixed TSPP–LaSurf complexes can spontaneously assemble into various nanostructures capable of binding the anticancer drug cisplatin. Morphological behavior, stability, and ability to drug binding of nanostructures can be tailored by varying the molar ratio and the concentration of components. The guest binding is shown to be additional factor controlling structural rearrangements and properties of the supramolecular TSPP–LaSurf complexes.

## 1. Introduction

The spontaneous organization of molecular building blocks relative to each other plays a huge role in the preparation of supramolecular and colloidal nanomaterials [1,2]. The self-assembly phenomenon inspires chemists and materials scientists to produce such materials with careful molecular organization control, without the use of lengthy synthetic procedures and organic solvents. A design of nanomaterials by this method has benefited to a large extent from cooperation between supramolecular and colloidal chemistry, which made it possible to design non-covalent complexes between hydrophilic and hydrophobic components, namely supramolecular amphiphiles or supra-amphiphiles. The emergence of interest in supra-amphiphilic systems is related to the fact that they can spontaneously assemble to create many well-defined nanostructures, which have enormous potential for application in drug and gene delivery, sensorics, protein recognition, and regulation of ion channels [3,4,5,6,7].

**Figure 1:**
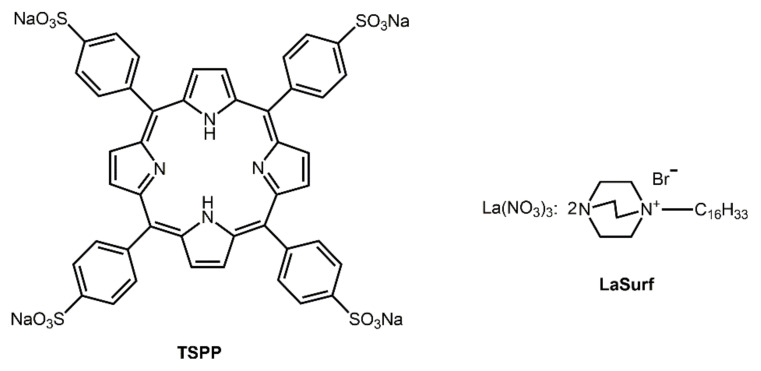
Chemical formulas of TSPP and LaSurf.

Currently, much attention in the field of drug delivery is paid to the search for self-assembling nanocontainers, showing targeted delivery into specific cells, drug protection from external influences, and increased therapeutic efficacy. The use for these purposes of supramolecular aggregates based on non-covalent host–guest complexes has a number of advantages, such as the simplicity of their preparation and modification with stimulus-sensitive and targeting ligands. A large number of works are devoted to the formation of mixed aggregates due to non-covalent interactions of macrocyclic hosts with open-chain surfactants and polymers [8,9,10,11]. Porphyrins are widely used as macrocycles in supramolecular aggregates due to their distinctive π-conjugated architecture, excellent photosensitivity, and biochemical functionality [12,13]. The ability of porphyrins and their metal complexes to accumulate in cancer tissues has been long-standing [14,15,16]. Due to this affinity for cancer cells and their photosensitizing properties, they are widely used in photodynamic therapy of cancer for visualizing the location of tumor foci in the body [14,17] and their destruction [16,18,19]. The combination of this effective and at the same time sparing method of cancer treatment with chemotherapy holds great promise for improving the quality of anticancer treatment [20]. The combined use of porphyrin and cisplatin induces apoptosis of HeLa cells upon laser irradiation [21]. Cisplatin, covalently bound to hematoporphyrin, exhibits selective cytotoxicity due to the affinity of porphyrins for tumor tissues [22]. Due to hydrophobic, electrostatic, and π–π stacking interactions, tetrasodium meso-tetra(sulfonatophenyl)-porphyrin (TSPP) (Figure 1) is able to form self-assembled nanoparticles with doxorubicin, which are destroyed in the tumor intracellular environment [23]. As a result of this drug release, doxorubicin can inhibit tumor proliferation, and TSPP upon light irradiation generates reactive oxygen species to sensitize multiresistant cells, which ultimately leads to apoptosis of tumor cells. In another work, pH-sensitive vesicles based on a porphyrin-containing polymer were obtained, which are also capable of solubilizing doxorubicin [24]. The resulting system exhibited high phototoxicity and significant tumor growth inhibition efficiency. Moreover, it is known that some types of porphyrins exhibit their own cytotoxic activity and can be used as chemotherapeutic agents [17,25].

The disadvantage of many porphyrins preventing their use in biomedicine is the spontaneous formation of aggregates in aqueous solutions due to the hydrophobic nature of the porphin core [26]. The processes of self-aggregation of porphyrins due to π–π interactions not only limit their targeted delivery to tumor cells, but can also lead to self-quenching in the excited state, which can reduce both the generation of reactive oxygen species and the toxic effect of the photosensitizer itself on tumor cells. The incorporation of porphyrins into supramolecular aggregates or attachment to various delivery vehicles is a strategy for improving and prolonging their functional activity and overcoming their limitations as therapeutic and diagnostic agents. An important role in such supramolecular self-organization is assigned to amphiphilic systems due to their high ability to form aggregates at sufficiently low concentrations. Mixed systems formed by non-covalent interactions between porphyrins and surfactants are widely researched. It is known that the interaction of porphyrin with surfactants in an aqueous medium leads to joint aggregation into various supramolecular structures, including macrocycle–surfactant complexes, J- and H-aggregates, and porphyrin-solubilized surfactant-based aggregates [27,28]. H-aggregates are dimers and larger aggregates of a columnar structure, in which porphyrin molecules are folded face-to-face, and in J-type aggregates the porphyrin molecules overlap with displacement relative to each other with the formation of a ladder structure [27,29]. Under this self-assembling strategy, much of the research was focused on the interaction of porphyrins with amphiphiles taken at concentrations above the critical micelle concentration, when macrocycle molecules are solubilized into the hydrophobic core of the micelles being formed [27,30,31,32]. Porphyrin molecules enclosed in micelles can fold into J-aggregates demonstrating chiro-optical properties [33,34]. In addition, if the porphyrin molecule has a charge, then the macrocycle may not solubilize into micelles of ionic surfactants, but cover their surface due to electrostatic interaction [35,36,37]. Much less emphasis is placed on the premicellar supramolecular porphyrin–surfactant complexes formed at amphiphile concentration that is considerably less than its critical micelle concentration [27,38,39]. The TSPP molecule forms complexes with cationic amphiphiles in a ratio of 1:4, in which four negatively charged groups of one porphyrin molecule are completely neutralized by four surfactant molecules. These complexes form monolayers at the interface, from which Langmuir–Blodgett films can be obtained [40,41,42]. Moreover, the supramolecular porphyrin–surfactant complexes in the bulk solution can assemble into spherical nanoparticles [39,43] and rod-like micelles which can transform into vesicular aggregates [44].

Since the aggregation of supramolecular porphyrin–amphiphile complexes in the solution is poorly studied, in this work we investigated the possibility of producing a non-covalent complex of TSPP with metalloamphiphile and the aggregating capacity of this complex in an aqueous medium. Hence, the objective of this study is to form supramolecular TSPP–metalloamphiphile complexes with emphasis on defining structural features of aggregates formed by them and demonstrating their ability to act as a nanocontainer for cisplatin. A dicationic surfactant was chosen as the metalloamphiphile, which is a complex of two fragments of diazobicyclooctane with hexadecyl tails and lanthanum nitrate (LaSurf). Lanthanum compounds show high antitumor activity [45,46,47] and are able to regulate the antitumor resistance of the organism [48]. The choice of a lanthanum-containing amphiphile is due to its ability to form joint aggregates with an oppositely charged 3D macrocycle, which can act as a medicinal nanocontainer of cisplatin with improved selectivity towards diseased cells [49]. To the best of our knowledge, there are no data in the literature concerning the complexation of porphyrin with metallosurfactant in the solution. Since the supramolecular amphiphiles TSPP–LaSurf exhibited the expected mixed aggregation ability, the aggregates based on the obtained supra-amphiphiles were used as nanocontainer for cisplatin. The choice of this anticancer drug is due to the revealed synergistic antiproliferative effect of photodynamic therapy using hematoporphyrin and cisplatin when acting on bladder cancer cells [50].

## 2. Materials and Methods

### 2.1. Materials

The metallosurfactant complex was synthesized according to the protocol reported in literature [51]. Pure surfactant (diazobicyclooctane with hexadecyl tail) was prepared according to a previously published procedure [52]. 5,10,15,20-tetrakis (4-sulfonatophenyl) porphyrin dodecahydrate (Alfa Aesar, Ward Hill, MA, USA, 95%), rhodamine B (Acros Organics, Fair Lawn, NJ, USA, ≥98%), and cisplatin (Sigma-Aldrich, St. Louis, MO, USA) were used as received. All solutions were prepared in deionized water (18.2 MΩ) obtained using a Millipore Direct-Q 5 water purification system.

### 2.2. Measurements

The absorption of solutions was measured using a Specord 250 Plus spectrophotometer (Analytik Jena, Jena, Germany) in quartz cuvettes. Fluorescence spectra were recorded using a Cary Eclipse G9800A fluorescence spectrometer (Agilent Technologies, Santa Clara, CA, USA) at 25 °C. The size of the aggregates was determined by dynamic light scattering on a Zetasizer Nano instrument (Malvern Instruments, Malvern, UK). The source of laser radiation was a He–Ne gas laser with a power of 4 mW and a wavelength of 632.8 nm. For zeta potential measurement the same instrument with laser Doppler velocimetry and phase analysis light scattering was used. The temperature of the scattering cell was maintained at 25 °C. The measurements were repeated at least three times. All scatter data were processed using Malvern Zetasizer 5.10 software (Malvern Instruments, Malvern, UK).

All NMR experiments were performed on a Bruker AVANCE(III)-500 spectrometer (Bruker BioSpin GmbH, Rheinstetten, Germany). The spectrometer was equipped with a Bruker multinuclear z-gradient inverse probe head capable of producing gradients with strength of 50 G cm^−1^. All experiments were carried out at 303 ± 0.2 K. Chemical shifts were reported relative to HDO (4.7 ppm) as an internal standard. The pulse programs for all NMR experiments were taken from the Bruker software library.

Microscopic analysis of samples was carried out in a transmission electron microscope Hitachi HT7800 (Hitachi, Tokyo, Japan). Sample preparation in the following way: 5 μL of 0.2 mM solution was placed on a formvar/carbon-coated copper grid (3 mm), drying was performed at room temperature. After drying the grid was placed in a transmission electron microscope. Analysis was held at an accelerating voltage of 100 kV in TEM mode.

### 2.3. Encapsulation of Rhodamine B

Rhodamine B-loaded aggregates were prepared as follows: rhodamine B was added to compositions of TSPP:LaSurf 1:1 and 1:2 (the concentration of TSPP varies from 0.025 mM to 0.2 mM) so that the concentration of rhodamine B was 0.02 mM. The unencapsulated rhodamine B was removed from the solution by dialysis (the pore size of the dialysis bag was 2000 Da) during the day. The amount of unloaded rhodamine B passed into external water was determined spectrophotometrically using a calibration curve (Figure S1). The encapsulation efficiency was determined using the following equation:

$$EE=\frac{m_{Rh}-m_{Rh-unloaded}}{m_{Rh}}\times 100\%$$

where *m_Rh_* is the mass of rhodamine B added, and *m_Rh-unloaded_* is the mass of unencapsulated rhodamine transferred into external water.

### 2.4. Cisplatin Complexation

After adding the deionized water to solid cisplatin, the concentration stock 1 mM cisplatin solution was sonicated for 10–15 min until complete drug dissolution. The mixed solutions were prepared by simply mixing certain amounts of aqueous stock solutions of cisplatin and TSPP–LaSurf complexes in the desired ratio.

### 2.5. Fourier Transform Pulsed-Gradient Spin-Echo NMR Measurements

The Fourier transform pulsed-gradient spin-echo experiments were performed by bipolar pulse pair-stimulated echo-longitudinal eddy current delay sequence. Data were acquired with a 50.0 and 75.0 ms diffusion delay, with bipolar gradient pulse duration from 1.8 to 3.6 ms (depending on the system under investigation), 1.1 ms spoil gradient pulse (30%), and a 5.0 ms eddy current delay. The bipolar pulse gradient strength was varied incrementally from 0.01 to 0.32 T/m in 16 steps. The temperature was set and controlled at 303 K with a 640 L/h airflow rate in order to avoid any temperature fluctuations owing to sample heating during the magnetic field pulse gradients.

The diffusion experiments were performed at least three times and only the data with the correlation coefficients of a natural logarithm of the normalized signal attenuation (ln I/I_0_) as a function of the gradient amplitude b = γ^2^δ^2^g^2^(Δ − δ/3) (γ is the gyromagnetic ratio, g is the pulsed gradient strength, Δ is the time separation between the pulsed-gradients, δ is the duration of the pulse) higher than 0.999 were included. Experimental data were processed with the Bruker TopSpin 3.2 software (Bruker BioSpin GmbH, Rheinstetten, Germany). The diffusion constants were calculated by exponential fitting of the data belonging to individual columns of the pseudo 2D matrix. Single components have been assumed for the fitting routine. All separated peaks were analyzed, and the average values were taken.

### 2.6. Cell Viability Evaluation

Cytotoxic effects on Chang liver and M-HeLa cells were estimated by means of the multifunctional a Cytell Cell Imaging system (GE Health Care Life Science, Uppsala, Sweden) using the Cell Viability Bio App which precisely counts the number of cells and evaluates their viability from fluorescence intensity data. The cells were plated into a 96-well plate at a concentration of 1 × 10^5^ cells/mL, 150 µL of medium per well and cultured in a CO_2_ incubator at 37 °C. After 24 h of seeding the cells into wells, the investigated TSPP–LaSurf or TSPP–LaSurf–cisplatin system was added at a preset dilution, 150 µL to each well. The dilutions of the system were prepared immediately in nutrient media. The value of IC_50_ (the drug concentration that inhibits cell growth by 50%) was calculated using Quest Graph™ IC_50_ Calculator (https://www.aatbio.com/tools/ic50-calculator, accessed on 1 March 2022). The experiments were repeated three times. Intact cells cultured in parallel with experimental cells were used as a control.

### 2.7. Hemolytic Activity

The toxicity was tested for hemolytic activities against human red blood cells (hRBC). Fresh hRBC collected from heparinized blood were rinsed 3 times with 35 mM PBS/0.15 M NaCl, pH 7.3 by centrifugation for 10 min at 800× *g* and resuspended in PBS. Test compound dissolved in PBS (concentrations 0.98–500 μg/mL) were then added to 0.5 mL of a stock solution of hRBC in PBS to reach a final volume of 5 mL (final erythrocyte concentration was 10% *v*/*v*). The resulting suspension was incubated under stirring at 250 rpm for 1 h at 37 °C. The samples were then centrifuged at 2000 rpm for 10 min. Release of hemoglobin from hRBC was monitored by measuring the supernatant absorbance at 540 nm. Controls for zero hemolysis (blank) and 100% hemolysis consisted of hRBC suspended in PBS and distilled water, respectively. The experiments were performed three times.

## 3. Results and Discussion

### 3.1. Spectrophotometry

**Figure 2:**
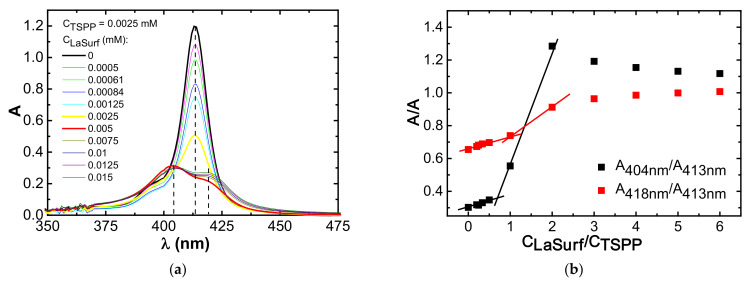
(**a**) Soret region of the absorption spectra of TSPP in the absence and presence of various amounts of LaSurf; (**b**) corresponding dependences of the ratio of absorption intensities (A_418nm_/A_413nm_, A_404nm_/A_413nm_) on the LaSurf fraction in a mixture with TSPP (H_2_O, 25 °C, 1 cm cell).

**Figure 3:**
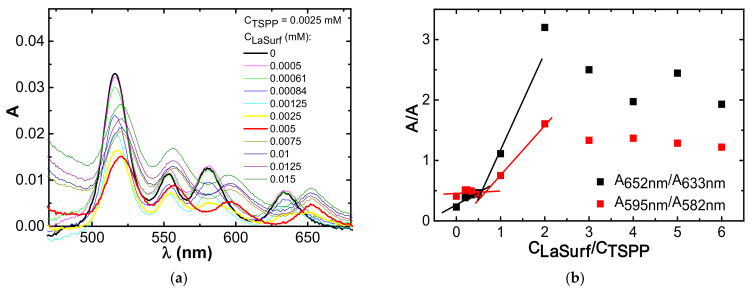
(**a**) Q band region of the absorption spectra of TSPP in the absence and presence of various amounts of LaSurf; (**b**) corresponding dependences of the ratio of absorption intensities (A_595 nm_/A_582 nm_, A_652nm_/A_633nm_) on the ratio of LaSurf amount in a mixture with TSPP (H_2_O, 25 °C, 1 cm cell).

UV spectra of 0.0025 mM TSPP solution in the absence and in the presence of various amounts of LaSurf are shown in Figure 2 and Figure 3. Considering the extensive literature data devoted to the study of the spectral properties of TSPP [27,38,43,53], it can be assumed that TSPP at a concentration of 0.0025 mM in an aqueous medium is in the form of a monomer, in which nitrogen atoms are not protonated, and sulfonate groups are dissociated to form four negatively charged groups. In the UV spectrum of pure TSPP, there is an intense Soret band at 413 nm (Figure 2a), as well as four distinguishable Q bands of comparatively lower intensity at 516 nm, 552 nm, 579 nm, and 634 nm (Figure 3a). This arrangement of bands in the UV and visible spectral regions corresponds to the monomeric tetraanionic state of TSPP.

The transition of porphyrin molecules from the monomeric form to H- or J-aggregates is easily detected spectrophotometrically, namely, during H- and J-arrangement of porphyrins the hypsochromic and the bathochromic shifts of Soret band are observed, respectively [27,29,32,37]. The UV spectrum of TSPP changes significantly when LaSurf is added to it in an aqueous medium. The intensity of the Soret band decreases with the addition of LaSurf until an equimolar amount is reached, i.e., up to a TSPP:LaSurf ratio of 1:1, and this band splits into two with maxima at 404 and 418 nm with a two-fold excess of LaSurf (Figure 2a). The appearance of absorption at 404 nm indicates a formation of H-type TSPP aggregates, and the presence of an absorption band at 418 nm can be caused either by the formation of mixed TSPP–LaSurf aggregates, in which the macrocycle is folded into J-aggregates [27,54,55] or by the inclusion of lanthanum in the porphyrin cavity [56]. Moreover, with a further increase in the amount of LaSurf in a mixed solution with TSPP, the absorption value at 404 nm slightly decreases, while that at 418 nm gradually increases. The dependences of the ratio of absorption intensities of bands at 404 and 418 nm relative to absorption value at 413 nm on the concentration ratio of LaSurf/TSPP were plotted (Figure 2b). It can be seen that the ratio of absorption intensities A_404nm_/A_413nm_ increases significantly when the equimolar ratio is reached, and the maximum on this dependence corresponds to a complex containing two LaSurf molecules and one TSPP molecule. At this composition of supramolecular complex, we can talk about the complete neutralization of four negative charges in TSPP by two pairs of positive LaSurf charges, as a result of which it is easier for the London dispersion forces between the porphin rings to overcome electrostatic repulsion, thus creating H-aggregates. A similar change is observed for the ratio of absorption intensities A_418nm_/A_413nm_, but after reaching a two-fold excess of LaSurf this ratio does not decrease, as in the case of A_404nm_/A_413nm_, and an increase in the proportion of LaSurf leads to the fact that the intensities of the absorption bands at 404 nm and 418 nm become close to each other. Probably, an increase in the concentration of LaSurf molecules leads to an enhancement of the hydrophobic effect, whereby the nanoparticles are formed in the form of joint aggregates, on the surface of which TSPP molecules are located. Most likely, the surface of such particles orients TSPP molecules through edge-to-edge into J-aggregates, and therefore the intensity of the absorption band at 418 nm increases, and the proportion of H-aggregates that give absorption at 404 nm gradually decreases, which probably indicates an equilibrium state of both types of aggregates.

As noted above, four Q bands appear in the absorption spectrum of single TSPP, which is characteristic of metal-free porphyrins due to reduced symmetry (Figure 3a). The initial addition of LaSurf to TSPP first causes a decrease in the absorption intensities of these bands. However, the absorption spectrum of the equimolar TSPP–LaSurf mixture in the visible region of the spectrum still has four weak absorption bands, which indicates the absence of incorporation of lanthanum atom of LaSurf into the coordinating cavity of TSPP. When LaSurf is added, all four Q bands of TSPP also undergo bathochromic shifts, which are likely due to changes in the microenvironment of the formed complexes. Possibly, the electrostatic compensation of negatively charged sulfonate groups TSPP with cationic fragments of LaSurf favors the attraction of TSPP molecules to each other with the formation of H-aggregates [57]. When a two-fold excess of LaSurf versus the TSPP is reached, the absorption spectrum of the mixed system changes significantly by the red shift of the Q bands, with the greatest shift observed for two Q bands in the long-wavelength region of the spectrum (from 582 nm to 595 nm and from 633 nm to 652 nm). A further increase in the LaSurf fraction leads to an increase in the absorption intensities of the Q bands, and the dependence of band intensity ratios in this long-wavelength region on the component ratio (LaSurf/TSPP) also shows the onset of the formation of a supramolecular complex at a 1:1 ratio and their rearrangement at a two-fold excess of LaSurf (Figure 3b). The obtained data can be correlated with the results of the work [58] which shows that an increase in the fraction of the counterion for anionic porphyrin causes the transition of H-aggregates to J-aggregates in accordance with the above discussion of spectral changes in the Soret band.

The corresponding changes in Soret and Q bands correlate with the measured pH values in aqueous solutions of 0.0025 mM TSPP in the absence (pH 7.63) and the presence of an equimolar amount (pH 7.36) and a two-fold excess of LaSurf (pH 7.08). A decrease in pH with an increase in the amount of LaSurf is caused by the presence of metalloamphiphile in the mixture, since the pH of an individual LaSurf solution with a concentration of 0.05 mM has a pH of 6.05. Nevertheless, the obtained pH values in mixed TSPP–LaSurf systems are higher than the pKa value of pure TSPP equal to 4.73 [59], which indicates the absence of protonated forms of the macrocycle in the complex with LaSurf. According to numerous literature data, the addition of an oppositely charged component to TSPP in a neutral medium causes the formation of H-aggregates [60,61,62]. Thus, after full electrostatic compensation of TSPP charges at TSPP:LaSurf = 1:2 ratio, an increase in the LaSurf fraction leads to an increase in the contribution of the hydrophobic effect to the formation of mixed aggregates. Taking into account the literature data, it can be assumed that after reaching a two-fold excess of LaSurf in the mixed system, with an increase in the LaSurf fraction, micellar aggregates begin to form, in which TSPP molecules either fold into J-aggregates on the micelle surface [36,37], or together with LaSurf complex are solubilized inside individual micelles [30,38,63].

### 3.2. Fluorescence Spectroscopy

**Figure 4:**
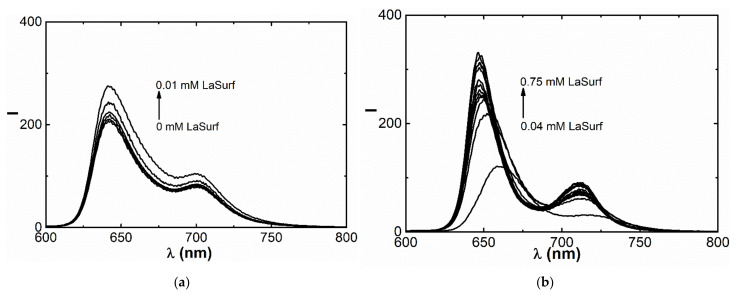

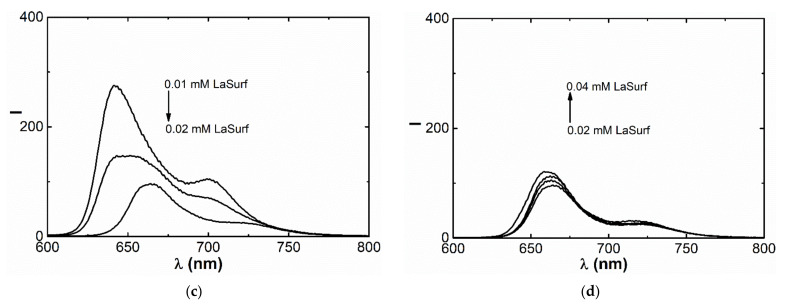
Fluorescence spectra of TSPP (0.01 mM) in the presence of (**a**) 0–0.01, (**b**) 0.01–0.02, (**c**) 0.02–0.04, and (**d**) 0.04–0.75 mM LaSurf.

Aggregation behavior in the TSPP–LaSurf system is also confirmed by experiments on TSPP fluorescence. The emission spectrum of pure TSPP in an aqueous medium shows two bands: more intense band at 644 nm and less intense of aggregates at 704 nm (Figure 4a), which confirms the dominance of the monomeric form of TSPP [27,38]. The addition of the first portions of LaSurf up to equimolar ratio leads to an increase in fluorescence (Figure 4a), which is probably caused by the formation of monomeric complexes, but not aggregates based on them [35]. Further increase of LaSurf concentration up to 0.02 mM quenches these fluorescence bands, which is caused by the mixed aggregation of TSPP molecules with amphiphilic molecules (Figure 4b) and correlates with the literature data [34,38,43,44]. It must be said that there are Coulomb repulsive forces between TSPP sulfo groups, which are compensated when interacting with LaSurf, as a result of which the aggregation of porphyrins only intensifies. At a charge ratio of TSPP:LaSurf = 1:2, the fluorescence intensity is the lowest, and the large red shift of both bands is observed associated with a change in the polarity of the surrounding porphyrin as a result of aggregation with LaSurf. The emission spectrum of this composition shows one emission maximum at 664 nm with a shoulder in the region of 720 nm, which corresponds to the formation of mixed aggregates. This shape of the spectrum is preserved up to a four-fold excess of LaSurf (Figure 4c). At higher concentrations of LaSurf the TSPP fluorescence sharply increases with blue shift of the emission maximum up to 647 nm, and a second maximum appears again at 710 nm (Figure 4d). The observed changes in this LaSurf concentration range are associated with the solubilization of porphyrin in the micellar surfactant medium [38,64].

Due to band shifts and nonmonotonic changes in TSPP emission intensities over a wide range of LaSurf concentrations, it is not possible to determine the porphyrin binding constant by metallosurfactant. Only after reaching a LaSurf concentration of 0.1 mM, a directly proportional increase in the TSPP fluorescence intensity is observed without a shift in the maximum (Figure 4d). Therefore, to calculate the binding constant, the concentration range from 0.1 mM to 0.75 mM corresponding to the micellar state of LaSurf was chosen. In this regard, a LaSurf concentration of 0.1 mM was defined as the critical concentration of co-aggregation in the presence of 0.01 mM TSPP and used to obtain the dependence (I/I_0_−1)^−1^ versus (C−cmc)^−1^ (Figure S2), from which the binding constant is defined as the ratio of intercept/slope values and equals 3161.5 M^−1^. This value of the binding constant can only be compared with compositions of metal-free and metal complexes of TSPP with ionic surfactants, since there are no works in the literature devoted to the complexation of nonmetallic porphyrin derivatives with metallosurfactants. Comparing with literature data [64,65], we can say that the efficiency of interaction between TSPP and metallosurfactant is at least an order of magnitude lower than in complexes of metal TSPP and cationic surfactants. A likely reason for the weaker interaction between TSPP and LaSurf is a steric mismatch between the various supramolecular interactions. So, the cationic groups of the diazobicyclo[2.2.2]octane (DABCO) fragment of LaSurf determine the affinity for the sulfo groups of TSPP, and the lanthanum fragment of LaSurf can be coordinated into the core of the porphyrin macrocycle. Most likely, these two points of contact between TSPP and LaSurf determine the dynamic nature of the intermolecular interaction between them, which is reflected in the reduced stability and binding constant.

### 3.3. NMR Spectroscopy

**Figure 5:**
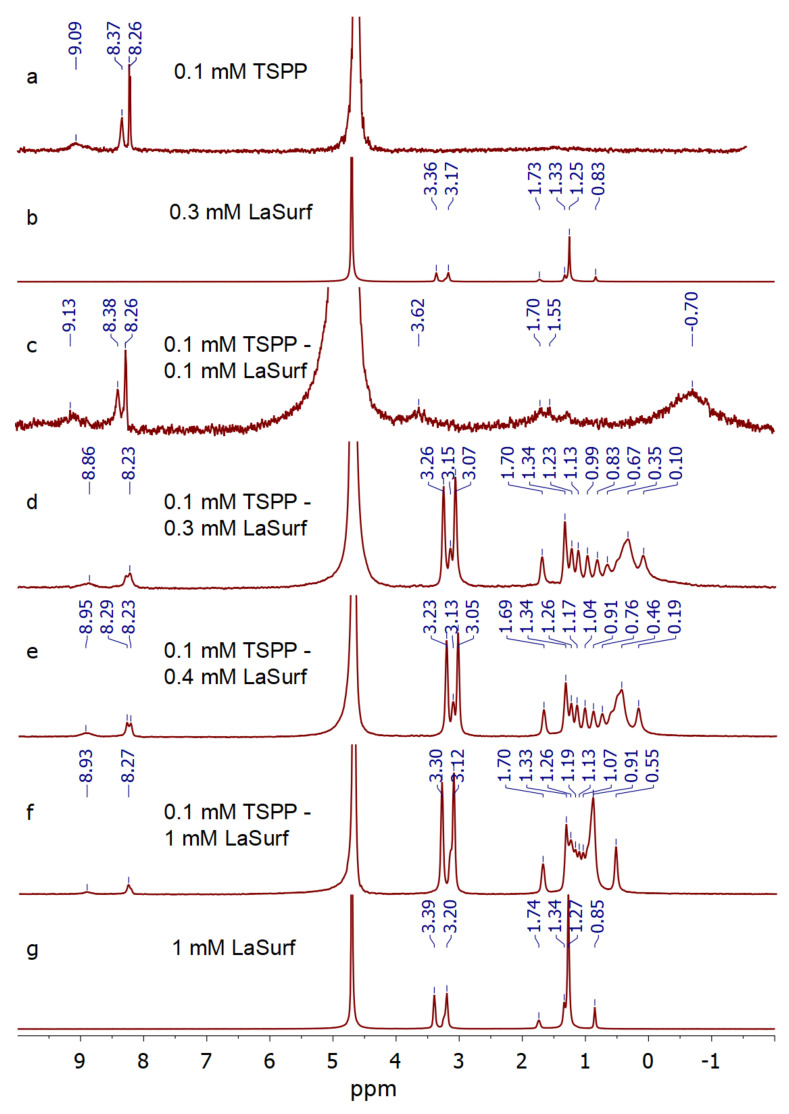
^1^H NMR spectra of pure (**a**) 0.1 mM TSPP, (**b**) 0.3, and (**g**) 1 mM LaSurf, as well as 0.1 mM TSPP in the presence of (**c**) 0.1, (**d**) 0.3, (**e**) 0.4, and (**f**) 1 mM LaSurf in D_2_O.

^1^H NMR spectra were obtained for TSPP in the absence and presence of various amounts of LaSurf, and the TSPP concentration in all samples was 0.1 mM. Strong complexation and subsequent joint aggregation in an equimolar 0.1 mM TSPP–0.1 mM LaSurf mixture leads to the disappearance of both TSPP and LaSurf resonances (Figure 5). A solution of 0.1 mM TSPP–0.2 mM LaSurf could not be studied due to the rapid formation of a precipitate; therefore, ^1^H NMR spectra were obtained and self-diffusion coefficients (D_s_) (Table 1) were measured for mixed compositions with three-, four-, and ten-fold excesses of LaSurf. The addition of an excess of LaSurf leads to an upfield shift of TSPP protons, with the largest chemical shift observed for the protons of sulfophenyl groups of TSPP, which indicates the electrostatic nature of the interaction between TSPP and LaSurf. It is very interesting to note that, in the spectrum of these mixtures, the signals of LaSurf alkyl chain clearly split into several separate resonances, in contrast to the spectrum of individual LaSurf, and the signals of methylene protons near the terminal methyl group are split most strongly (Figure 5), which indicates the interaction of the porphyrin with the hydrophobic fragments of LaSurf [66,67]. The most significant high-field shift in this methyl group in a mixture with a three-fold excess of LaSurf compared to mixtures with a four- and ten-fold excess confirms the strong complexation in the mixture near the stoichiometric ratio TSPP:LaSurf = 1:2. Furthermore, a separate resonance appears between the signals of the protons of DABCO fragments at ~3.15 ppm, which is probably related to the so-called DABCO sandwich resonance as a result of being surrounded by two TSPP molecules [68]. In this sandwich the methylene units of hexadecyl tails of LaSurf, depending on the location between two TSPP molecules, undergo different high-field displacements. According to the effect of magnetic anisotropy of the porphyrin macrocycle, the data obtained indicate that the long alkyl chains of LaSurf in the composition of the ion complex with TSPP are located below the porphyrin plane [44,69,70]. The location of the LaSurf alkyl chains under the TSPP plane is possible due to hydrophobic effect stabilized by CH–π interactions between the methylene protons of LaSurf and the π-system of the porphyrin macrocycle. Consequently, the LaSurf molecule structures around the TSPP molecule due to the cooperative effect of supramolecular interactions and folds into sandwich-like structures. Taking into account the UV spectra of the TSPP mixture with a two- or more-fold excess of LaSurf, it can be assumed that these sandwiches mainly form H-aggregates as a result of intermolecular interactions between TSPP, the proton signals of which in the complex with LaSurf are broadened in ^1^H NMR spectra. An increase in the fraction of LaSurf from 0.3 mM to 1 mM in a mixture with TSPP leads not only to a decrease in the broadening of the signals of the protons of the alkyl chains, but also to a decrease in the number of splitting signals near the terminal methyl group, which indicates a possible additional aggregation of the alkyl chains of LaSurf with each other. At the same time, the upfield shift of the alkyl protons of LaSurf mixed in a ten-fold excess with TSPP decreases, and there is a much less clear pattern of the set of peaks compared with mixture 0.1 mM TSPP–0.3 mM LaSurf due to the contribution of probably individual LaSurf aggregates. It is also worth noting the disappearance of the signal between the methylene protons of DABCO and the broadening of the signals of the porphyrin protons observed in a mixture with a ten-fold excess of LaSurf, which can probably be due to an increase in the contribution of J-aggregates [71]. Thus, summing up the data of spectrophotometric titration and NMR spectroscopy, we can say that stoichiometric complexes are capable of aggregating into H-aggregates, in which TSPP molecules are sandwiched, and aliphatic chains are located between them. An increase in the proportion of LaSurf in a mixture with TSPP (>1:2 mole ratio) leads to the fact that additional binding of LaSurf is likely to occur between porphyrin molecules, which possibly changes the orientation of TSPP molecules from H- to J-aggregation.

According to the data given in [72], as the pH increases by 1, the HDO (4.7 ppm) chemical shift used as an internal standard changes upfield by 0.002 ppm. In our case, as the LaSurf concentration increases up to 1 mM, the pD changes from 6.92 to 5.87, and the observed changes in chemical shifts in TSPP–metallosurfactant significantly exceed 0.002 ppm. Therefore, the addition of LaSurf changing the chemical shift in solutions studied by ^1^H NMR does not distort the conclusions made. To further confirm these conclusions, the self-diffusion coefficients (D_s_) were further measured by the Fourier transform pulsed-gradient spin-echo NMR [73], where there is no need for high-precision determination of chemical shifts. This technique showed that the D_s_ values of TSPP and LaSurf in mixtures are lower than those for free TSPP (3.35 × 10^−10^ m^2^ s^−1^) and free LaSurf that is even in an individual aggregated form at a concentration of 1 mM (1.44 × 10^−10^ m^2^ s^−1^), which confirms the occurrence of strong joint aggregation of both compounds (Table 1). The D_s_ values for TSPP in a mixture with different amounts of LaSurf is kept at the level of 0.8 × 10^−10^ m^2^ s^−1^, which probably corresponds to the Ds value of the joint complex. The Ds value of LaSurf is minimal (1.31 × 10^−10^ m^2^ s^−1^) in a system with a three-fold excess, which means its maximal inclusion in mixed aggregation with TSPP in this mixture compared to a mixture with a four-fold excess of LaSurf, for which the Ds value slightly increases to 1.46 × 10^−10^ m^2^ s^−1^. It could be assumed that such an increase in the Ds value for LaSurf indicates that a large excess of LaSurf molecules probably does not find a place for binding in the TSPP–LaSurf complex, and an excess of LaSurf leads to the formation of own LaSurf aggregates, which is reflected in a decrease in the Ds value up to 1.36 × 10^−10^ m^2^ s^−1^ with a ten-fold excess of LaSurf. This change of Ds value for LaSurf is a reliable indication that the presence of an excess of LaSurf in the solution does not change the stoichiometry of TSPP–LaSurf complexes. However, it should not be ruled out that an excess of LaSurf may initiate morphological rearrangement of some of the H-aggregates into J-aggregates, which follows from the spectrophotometric titration data (Figure 2 and Figure 3). An increase in the proportion of LaSurf in the mixture, accompanied by a decrease in its Ds to 1.36 × 10^−10^ m^2^ s^−1^, presumably means the enlargement of individual LaSurf aggregates, solubilizing TSPP–LaSurf complexes, in which porphyrin molecules are possibly packed into J-aggregates.

### 3.4. Transmission Electron Microscopy and Dynamic Light Scattering

**Figure 6:**
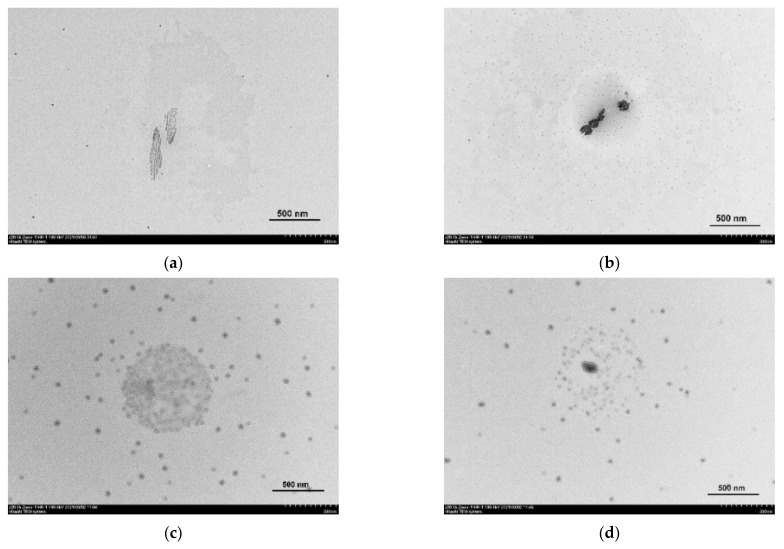
TEM images of TSPP in the absence (**a**) and presence of an equimolar amount (**b**), a two-fold (**c**), and three-fold (**d**) excess of LaSurf.

Transmission electron microscopy (TEM) was used to determine the morphology of nanoparticles formed by TSPP in the absence and presence of different amounts of LaSurf. TEM investigations were launched with pure TSPP solution, and the TEM image of this solution shows large columnar structures (Figure 6a). In addition, this image for free TSPP shows the presence of a small number of distinct structures of much smaller size. By comparison, the technique of particle analysis using dynamic light scattering (DLS) yields only the particles, the size of which corresponds to the size of an individual TSPP, but the polydispersity index is quite high (Table S1). The discrepancy between the sizes determined by TEM and DLS, namely the absence of large particles on the DLS size distribution, is probably due to the agglomeration of TSPP molecules on the grid during sample preparation. The addition of an equimolar amount of LaSurf to TSPP significantly increases the number of small particles and results in the presence of large aggregates in the TEM image (Figure 6b). Moreover, these aggregates in the equimolar mixture became much darker than in the image for free porphyrin, which probably indicates their adhesion under the action of LaSurf capable of complexing with TSPP due to electrostatic interaction and enhancing intermolecular interactions of the formed complexes due to hydrophobic interactions. A significant enlargement of aggregates in an equimolar composition is also observed in DLS distribution of hydrodynamic diameter in all three parameters (by intensity, by volume, and by number) (Table S1).

A very interesting TEM image was obtained for a mixed composition with a two-fold excess of LaSurf (Figure 6c). This image shows fairly widely distributed small spherical aggregates and concentrated large spherical aggregate. The supposed reason for the formation of such a picture is the fragmentation of aggregates as a result of effective electrostatic interaction of TSPP with LaSurf. When four negative charges of one TSPP are fully compensated by four positive charges of two LaSurf, supra-amphiphilic complexes are formed, which aggregate into small spherical micelles. The formed micellar particles are covered with macrocycles, which is in good agreement with the data of another work [44], and in the composition of these micellar aggregates the TSPP molecules are arranged in J-aggregates that are driven by edge–edge hydrophobic interactions, which correlates with the obtained spectrophotometric results (Figure 2a). As is known, micellar nanoparticles are rather unstable structures due to dynamic exchange between the micelle and the free monomers from solution. Probably, a peculiar large aggregate, presented in the center of the TEM image (Figure 6c), is formed from supra-amphiphiles based on TSPP and LaSurf, stacked as a result not only of hydrophobic interaction of alkyl chains of LaSurf in the composition of supra-amphiphiles, leading to the formation of joint micellar particles, but of H- and/or J-aggregation of TSPP fragments in the composition of supra-amphiphiles. The light coils observed in this aggregate are most likely these TSPP fragments, since they do not contain metal, the presence of which usually causes darkening in TEM images [74,75]. Thus, the presence of a two-fold excess of LaSurf in an aqueous medium of TSPP leads to morphological rearrangement of TSPP molecules with the formation of TSPP–LaSurf supra-amphiphiles, which can aggregate into small micelles due to micellization with the participation of LaSurf part of supra-amphiphiles, as well as into a large aggregate due to the H- and/or J-aggregation of TSPP part of supra-amphiphiles.

A further increase in the proportion of LaSurf in a mixture with TSPP to a four-fold excess reduces the proportion of supra-amphiphiles in the composition of a large aggregate (Figure 6d). It should be noted that, along with this, a slight increase in the diameter of micellar particles is observed. It is especially interesting to distinguish a rather large (relative to micelles) aggregate covered with light TSPP ‘balls’ with a darkened center, associated with the presence of an inorganic lanthanum component. In general, the TEM images obtained confirm the supramolecular behavior between TSPP and LaSurf in an aqueous medium revealed during spectrophotometric titration (Figure 2 and Figure 3), and it can be confidently asserted that the complexation between them leads to the formation of nanoparticles, the structure of which is determined by the charge stoichiometry.

### 3.5. Aggregation Stability of Stoichiometric TSPP–LaSurf Complexes

**Figure 7:**
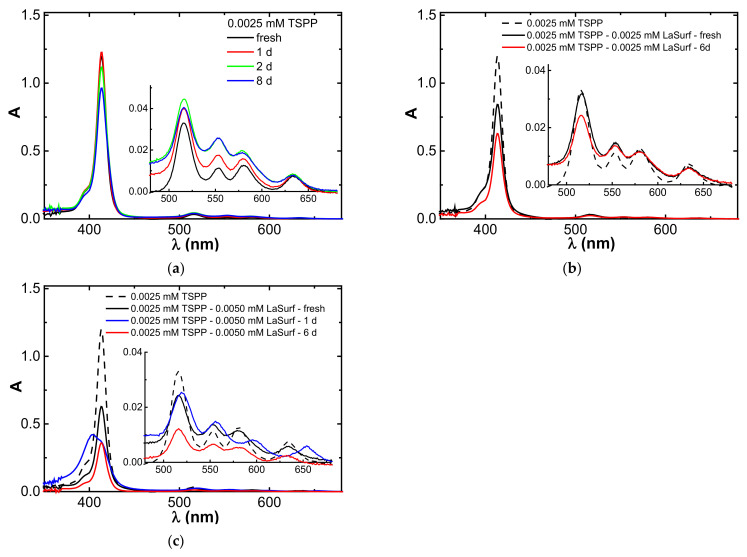
Changes of UV spectra of the Soret band and Q bands (inset) of pure 0.0025 mM TSPP (**a**) and mixed systems TSPP:LaSurf = 1:1 (**b**) and 1:2 (**c**) with time (H_2_O, 25 °C, 1 cm cell).

It is well known that the TSPP monomers are unstable over time due to the aggregation ability [76], and when stored at room temperature the intensities of Soret bands of individual TSPP decrease over time, and the absorption of its Q bands increases (Figure 7a). A similar spectral change in the Soret band is observed in the equimolar TSPP–LaSurf mixture, but the increase in the absorption intensities of the Q bands in the spectrum of this mixture is not as significant as in the case of pure TSPP (Figure 7b). Moreover, the longer the wavelength of the Q band, the less the increase in the intensity of its absorption in an equimolar mixture over time. This is probably due to the fact that, in the mixed equimolar TSPP–LaSurf system, TSPP molecules are preorganized in the presence of LaSurf, which slows down the breaking of the symmetry of the TSPP ring in time.

An interesting observation arose during storage of a mixed system with a two-fold excess of LaSurf: if the spectrum of a freshly prepared solution had a split Soret band, then the next day this Soret band again acquired a single peak, but with a lower intensity relative to the initial spectrum of individual TSPP, and the absorption of this band also decreased during time without subsequent splitting (Figure 7c). Two long-wavelength Q bands in the spectrum of a mixture with a two-fold excess of LaSurf undergo hypsochromic shifts the next day, and their intensity decreases with time. These spectral changes are likely related to the dynamic nature of the driving forces of aggregation between LaSurf and TSPP. The addition of two dicationic LaSurf molecules to one tetraanionic TSPP molecule first initiates the formation of H-aggregates, detected by the appearance of absorption at 404 nm. The next day, these H-aggregates spontaneously rearrange into monomeric complexes, which is due to the opposite appearance of an absorption band with a maximum at 413 nm. Thus, the stability of a mixed system with a two-fold excess of LaSurf is lower than in an equimolar system when stored at room temperature and in daylight, and this instability is due to the labile aggregation behavior of TSPP–LaSurf complexes.

Further, a comparative study of the concentration stability of aggregates formed from stoichiometric complexes TSPP:LaSurf = 1:1 and 1:2 was carried out. First, aggregation in the TSPP–LaSurf system was investigated spectrophotometrically by the change in absorption depending on the concentration of the supramolecular complex. In the TSPP:LaSurf = 1:2 system, the absorption at 404 nm increases with an increase in the concentration of the system (Figure S3), however, already at 0.01 mM TSPP–0.02 mM LaSurf, the aggregates formed in the solution precipitate, which introduces an error in obtaining the UV spectrum. In this regard, the concentration dependence of the change in the absorption at 404 nm for the TSPP:LaSurf = 1:2 system has a nonlinear character (Figure S3). The deposition of a precipitate in a highly concentrated solution is explained by the low solubility of the complex due to the complete neutralization of the charges of the ionic components, which promotes the formation of joint hydrophobic aggregates.

**Figure 8:**
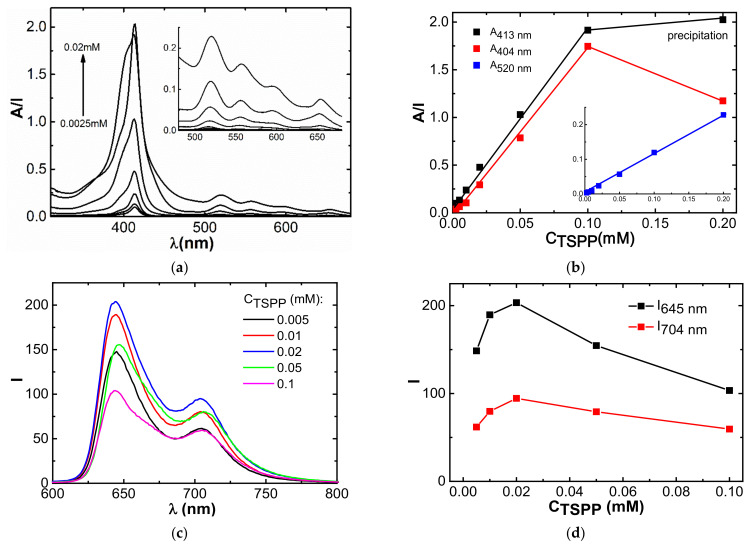
Absorption (**a**) and fluorescence (**c**) spectra and corresponding dependences of absorption at 404, 413, and 520 nm (**b**) and fluorescence intensity at 655, 704 nm (**d**) versus concentration of TSPP:LaSurf = 1:1 mixture. The insets detail changes of UV spectra of Q bands (**a**) and concentration dependence of absorption at 520 nm in TSPP:LaSurf = 1:1 mixture (**b**).

Due to the low stability of the TSPP:LaSurf = 1:2 composition, we further investigated a system with a 1:1 ratio, in which half of the negative charges in the TSPP molecule remain uncompensated by the cationic LaSurf fragments. The complexes formed in this equimolar system are more stable, since the precipitate begins to form only at component concentrations above 0.2 mM; therefore, starting from this concentration, the dependence of the absorption of the Soret band loses its linear form (Figure 8b). In addition to an increase in the absorption of the Soret band at 413 nm, an increase in the absorption of the shoulder of this band at 404 nm is observed with an increase in the concentration of the equimolar TSPP–LaSurf mixture to a concentration of 0.2 mM (Figure 8a). Taking into account the literature data [27,76], the appearance of a shoulder at this wavelength indicates the presence of H-aggregates corresponding to the face-to-face stacking. However, at a concentration of 0.2 mM in an equimolar mixture, the shoulder of the Soret band disappears, which indicates the decomposition of H-aggregates into monomeric complexes, an increase in the concentration of which leads to precipitation. Interestingly, the absorption of the Q bands increases in direct proportion with the concentration of TSPP:LaSurf = 1:1 in a fairly wide concentration range up to 0.2 mM, which indicates the retention of the symmetry of the porphyrin fragment in the complex with LaSurf during this rearrangement of the H-aggregates (Figure 8b). Nevertheless, a study of the emissivity of TSPP:LaSurf = 1:1 complexes showed that a different concentration is critical for this equimolar system. The fluorescence intensities of the two characteristic TSPP bands first increase with an increase in the concentration of the components and then decrease after the inflection observed for 0.02 mM TSPP–0.02 mM LaSurf composition (Figure 8c,d). The increase in emission is caused by an increase in the amount of both monomeric TSPP–LaSurf complexes, while the decrease in fluorescence intensity observed after 0.02 mM is probably related to the self-quenching of TSPP due to the aggregation of the complexes.

### 3.6. Encapsulation of Rhodamine B

During measurements of the size of aggregates formed in TSPP–LaSurf solutions using DLS, the hydrodynamic diameter values with a wide range from 30 nm to 430 nm are recorded (Table S1, Figure S4), indicating a high polydispersity of the systems. Nevertheless, when measuring these sizes, a good correlation function is formed, which indicates the spherical shape of the aggregates. Considering the extensive literature data of mixed TSPP compositions with other amphiphilic components [27,28,29,38,39,43,44] and the spectral changes obtained in this work, it can be concluded that H- and J-aggregates are formed in the TSPP–LaSurf system, the proportion of which depends on the LaSurf:TSPP ratio. Based on the data obtained by TEM, DLS, spectrophotometry, and fluorescence, the TSPP:LaSurf = 1:1 and 1:2 complexes form different aggregates in an aqueous medium. Since these aggregates may contain a hydrophilic core, the ability of TSPP:LaSurf aggregates with a ratio of 1:1 and 1:2 to encapsulate a model hydrophilic rhodamine B probe was further investigated.

In the TSPP:LaSurf = 1:2 system, a two-fold increase in the concentration of the components leads to an increase in encapsulation efficiency (EE%) of rhodamine B from 5 to 38% (Figure S5). A further two-fold increase in the concentration (up to 0.01 mM TSPP–0.02 mM LaSurf) causes the precipitation, and the EE% value decreases due to this instability. Aggregates of a more stable equimolar composition with an increase in concentration to 0.1 mM do not precipitate and, as a consequence, encapsulate more dye (EE% of 55%). The system 0.2 mM TSPP–0.2 mM LaSurf is stable and does not even opalesce in an aqueous medium, but if rhodamine B is added to it, a precipitate occurs within 1 h. Interestingly, during the extraction of unencapsulated rhodamine B from this solution, only about 20% of free rhodamine B escaped into the water outside the dialysis bag. Hence, the rhodamine B molecule forms a ternary complex with the equimolar TSPP–LaSurf system, in which two free sulfonate groups of TSPP can electrostatically interact with the cationic form of rhodamine B. This charge–charge interaction between rhodamine B and TSPP causes a complete compensation of all negative charges of TSPP, resulting in the precipitation of the ternary complex. Thus, despite the strong complexation of TSPP with LaSurf, the macrocycle molecule retains its binding capacity to the third component.

**Figure 9:**
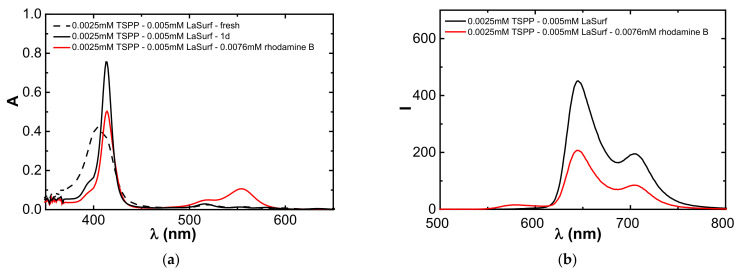

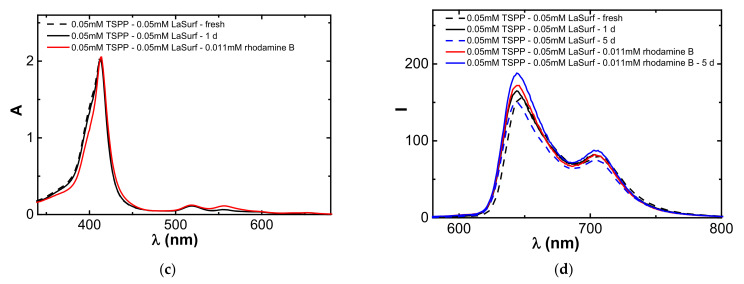
UV (path lengths of 10 mm (**a**) and 2 mm (**c**)) and fluorescence spectra (**b**,**d**) for TSPP:LaSurf = 1:2 and 1:1 systems with encapsulated rhodamine B.

As can be seen from Figure 9a, for TSPP:LaSurf = 1:2 system after the release of unencapsulated rhodamine B during dialysis, a bathochromic shift of the Soret band is observed in the absorption spectrum, which is probably related with the aggregation of the ternary complex. At the same time, the fluorescence intensity at the 644 and 704 nm is immediately strongly reduced (Figure 9b). The addition of rhodamine B to equimolar system insignificantly affects the UV spectra of TSPP–LaSurf compositions and only an increase in the absorption band at 555 nm corresponding to the dye (Figure 9c). Also, in contrast to the complex with a double excess of LaSurf, the addition of rhodamine B to the equimolar composition leads to a slight increase in the fluorescence intensity, which increases even more within 5 days due to the prolonged release of encapsulated rhodamine B (Figure 9d). The different fluorescent behavior of binary TSPP:LaSurf systems in ratios 1:1 and 1:2 in the presence of rhodamine B once again points to the greater stability of the equimolar system, the emission spectrum of which does not undergo significant changes after the encapsulation of rhodamine B in time.

### 3.7. Complexation with Cisplatin

**Figure 10:**
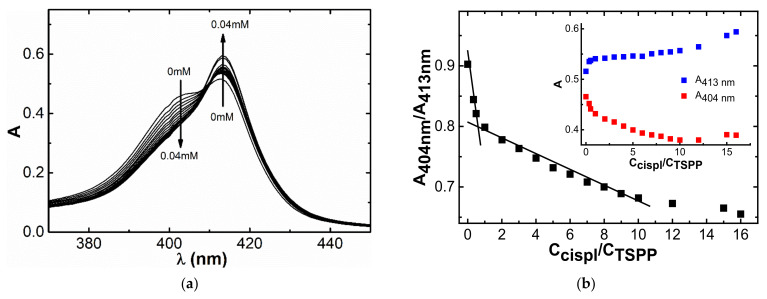
(**a**) Changes in UV spectra of 0.0025 mM TSPP–0.005 mM LaSurf system with increasing cisplatin concentration and (**b**) corresponding dependences of the ratio of absorption intensities (A_404nm_/A_413nm_) on the cisplatin concentration. The inset shows dependences of absorption intensities at 404 nm and 413 nm in 0.0025 mM TSPP–0.005 mM LaSurf on the cisplatin concentration.

The next step was to evaluate the binding of TSPP–LaSurf systems of the anticancer drug cisplatin. The decision to choose such a toxic drug is stipulated both by the low cost as compared with doxorubicin hydrochloride and the absence of hemolysis and cytotoxicity against Chang liver cells of mixed TSPP–LaSurf systems, regardless of the amount of LaSurf (Tables S3 and S4). Despite the presence of hydrophobic fragments (porphin core of TSPP and alkyl chains of LaSurf), all compositions showed a low hemolytic coefficient, which confirms good blood compatibility. The absence of hemolysis is probably due to the hydrophilic nature of the formed supramolecular systems in a rather low concentration range. When cisplatin is added to a solution containing 0.0025 mM TSPP and 0.005 mM LaSurf, a decrease in absorbance at 404 nm and an increase in absorbance at 413 nm are observed (Figure 10a). The plotted dependences of the values of the absorption intensities at these wavelengths show a change in the slope at the concentration of cisplatin equimolar to that of TSPP (Figure 10b), which suggests that drug binding is due to the macrocyclic component of the complex. The decrease in absorption at 404 nm with an increase in the proportion of cisplatin is probably caused by the rearrangement of H-aggregates under the action of the drug. Presumably, H-aggregates in a solution containing 0.0025 mM TSPP and 0.005 mM LaSurf are destroyed, which is indicated by a change in the hydrodynamic diameter upon addition of cisplatin. DLS method showed that the presence of an excess of cisplatin leads to an increase in the size of the aggregates (Table S1, Figure S4). Taking into account the spectrophotometric decrease in the band at 404 nm, which corresponds to the proportion of H-aggregates, and the increase in the intensity of the Soret band upon the addition of cisplatin, it can be assumed that the presence of cisplatin in the TSPP:LaSurf = 1:2 complex leads to the destruction of H-aggregates of these complexes. Those, in turn, rearrange into larger aggregates of a different morphology, which resulted in an increase in the polydispersity index. It should also be noted that the presence of cisplatin in 0.0025 mM TSPP–0.005 mM LaSurf mixture also causes a change in the form of the correlation function, which possibly indicates a violation of the spherical shape of 0.0025 mM TSPP–0.005 mM LaSurf aggregates in the presence of cisplatin. The interaction of these aggregates with cisplatin is also confirmed by an increase in the zeta potential towards positive values from −17.0 mV (in the absence of cisplatin) to +4.5 mV (in the presence of cisplatin). Taking into account the observed changes in the shape of absorption bands, the size of aggregates, and the zeta potential in a 0.0025 mM TSPP–0.005 mM LaSurf solution, it can be assumed that two LaSurf molecules electrostatically interact with four sulfonate groups of one TSPP molecule. In this case, the lanthanum atom of LaSurf does not coordinate with the TSPP porphine core due to the strong electrostatic attraction of DABCO fragments, neighboring lanthanum, to TSPP sulfonate groups, which distances lanthanum from the porphyrin fragment and allows macrocycles to fit into H-aggregates. However, cisplatin molecules are able to destroy these aggregates by interacting with the TSPP porphyrin ring probably being located between two porphyrin planes of mixed aggregates. This statement is supported by the literature data, according to which metalation of free ligands is accompanied by the hyperchromic effect of the Soret band [77,78,79] and a more significant decrease in the intensity of free TSPP emission with a two-fold excess of the TSPP than with other ratios of TSPP:cisplatin (Figure S6a). It should be noted that the intensity of Soret band of pure TSPP the absence of LaSurf undergoes a less significant hyperchromic effect compared to binary TSPP–LaSurf system (Figure S7), and the lack of interaction between single TSPP and cisplatin is also confirmed by NMR spectroscopy (data not shown). Therefore, the electrostatic compensation of TSPP sulfonate groups by LaSurf cationic fragments favors the interaction of cisplatin with the porphyrin fragment in the complex with metallosurfactant.

The picture is quite different when cisplatin is added to the equimolar system. In the UV spectra of this system by the addition of cisplatin, the absorption at 413 nm does not increase as sharply as in the case of a mixed system with a two-fold excess of LaSurf. After reaching the amount of cisplatin equimolar to TSPP, the intensity of the Soret band of TSPP:LaSurf = 1:1 complex also slightly decreases (Figure S8a), and the presence of breakpoint at a cisplatin concentration equimolar to TSPP again highlights that a macrocyclic moiety is involved in drug binding (Figure S8b). Therefore, unlike the TSPP:LaSurf = 1:2, the view of the Soret band, the values of zeta potential (Figure S9), hydrodynamic diameter, and polydispersity index (Table S1) for the equimolar system do not undergo significant changes upon the addition of cisplatin. Approximately the same zeta potential value for TSPP:LaSurf = 1:1 and TSPP:LaSurf = 1:2 systems is due to the mobility of sulfophenyl fragments TSPP [55], which are probably able to cover the dicationic fragment of one LaSurf molecule in an equimolar mixture, thereby screening the positive charge. This equimolar TSPP–LaSurf complex is capable of aggregating into spherical aggregates, the stability of which is higher than that of aggregates formed in a solution with a two-fold excess of LaSurf. The stability of aggregates of the equimolar system in comparison with a mixture with a two-fold excess of LaSurf is also manifested in the ability to cisplatin binding. The addition of cisplatin to TSPP:LaSurf aggregates taken in 1:2 ratio causes a significant increase in their size and polydispersity, while the presence of cisplatin in the equimolar mixture practically does not affect the size of aggregates formed from these complexes. It is very interesting that when studying the effect of cisplatin on the emission of mixed TSPP:LaSurf compositions at different ratios (1:1 and 1:2), there was no significant change in emission intensities (Figure S10). The fluorescence spectrum of the TSPP–LaSurf mixture in the presence of cisplatin slightly differs from the spectrum of the free mixture. Thus, drug binding by TSPP–LaSurf compositions does not significantly affect the electronically excited levels of TSPP, which is an important factor for using the obtained supramolecular complex as a photosensitizer.

The effect of cisplatin on supramolecular TSPP–LaSurf system was also studied using NMR spectroscopy. The ^1^H NMR spectra of mixtures of 0.1 mM TSPP with different amounts of LaSurf (0.1 mM, 0.3 mM, and 1 mM) were compared in the absence and presence of cisplatin (Figure S11). The addition of an equimolar amount of cisplatin to TSPP:LaSurf = 1:1 system gives rise to a new signal in the region of 1.5 ppm, which also appears in a mixture with a three-fold excess of LaSurf. However, in a mixture with a large excess of LaSurf (0.1 mM TSPP–1 mM LaSurf–0.1 mM cisplatin), no such changes were observed after the addition of cisplatin. In the NMR spectra of ternary systems with 0.1 and 0.3 mM LaSurf, the appearance of a new signal between the signal of methylene protons of alkyl chains near a positively charged nitrogen of LaSurf and the signal of following protons of these chains can be related both with the formation of a new bond of these complexes with cisplatin and with the fact that the methylene groups of alkyl chains of LaSurf can additionally undergo a shielding effect due to the action of cisplatin, with the exception of the protons that are near the polar head of DABCO and the protons of DABCO fragments themselves. It can be assumed that cisplatin partially displaces LaSurf from the mixed TSPP–LaSurf complex, but the LaSurf molecule remains in the complex with TSPP, which is confirmed by the absence of significant changes in the self-diffusion coefficients of TSPP and LaSurf in ternary systems with cisplatin (Table S2). The presence of cisplatin in a mixture of TSPP with a ten-fold excess of LaSurf leads to the fact that it becomes more profitable for the excess of LaSurf molecules to escape into the solution volume and form their own individual aggregates, and, therefore, the addition of cisplatin does not cause such significant changes in the ^1^H NMR spectra as in the case of a lower excess LaSurf.

Needless to say, the exact location of cisplatin in the TSPP–LaSurf complex requires a separate careful study. Taking into account spectrophotometric data, the presence of four Q bands of TSPP in complexes with LaSurf indicates the retention of symmetry of the porphyrin macrocycle after addition of cisplatin (Figure S12). Therefore, it can be assumed that the driving force behind the binding of cisplatin by TSPP–LaSurf complexes is most likely not the coordination of the platinum atom with the porphin core of TSPP, but the hydrogen bonding between the ammonium groups of the drug and the pyrrole nitrogen of the macrocycle [80]. This interaction of cisplatin with TSPP–LaSurf complexes probably leads to disruption of the H-orientation of TSPP–LaSurf layers.

### 3.8. Cytotoxic Effect of Cisplatin in TSPP–LaSurf Compositions

Before proceeding to the study of the cytotoxicity of encapsulated cisplatin, the stability of TSPP–LaSurf compositions in the absence and presence of cisplatin under physiological conditions, namely in the cell culture medium, was studied. When comparing the emission spectra of these compositions in aqueous and cellular media, the emission bands in the cellular medium are shifted to the red region (Figure S13a), which is characteristic for porphyrin compositions [81]. This bias is caused by the presence of fetal bovine serum required to stabilize the cell environment (Figure S14a). It is interesting that a significant change in the form of the spectrum is observed for TSPP:LaSurf = 1:2 composition, which is associated with the disaggregation of H-aggregates into monomers in the cellular medium. The observed disaggregation in the composition TSPP:LaSurf = 1:2 is also caused by the presence of serum, since the appearance of the emission spectrum of TSPP:LaSurf = 1:2 in an aqueous solution is similar to that observed in a cell medium without serum (Figure S14b). To track the stability of the compositions, the change in the intensity of the emission band at 650 nm was monitored for 13 days, and the fluorescence intensity of all studied compositions slightly increased by ~200 au over the first two days, which is associated with an increase in the proportion of the monomeric form of TSPP in the cellular medium. During the remaining 11 days, the change in fluorescence was negligible (Figure S13b), which indicates good stability of TSPP–LaSurf in the absence and presence of cisplatin.

Since titration of 0.0025 mM TSPP–0.005 mM LaSurf complex with cisplatin revealed the first inflection at the amount of drug equimolar to the amount of the macrocycle, the cytotoxic effect on Chang liver and cancer M-HeLa cell lines at various concentrations of LaSurf were first studied. As can be seen from the Table 2, the presence of cisplatin in the equimolar complex leads to a sharp appearance of the cytotoxic effect, and taking into account the literature data of cytotoxicity for pure cisplatin in water [49], we can say the same about the manifestation of the cytotoxic effect of the triple complex. An increase in the proportion of LaSurf in this ternary complex insignificantly changes the cytotoxicity to both cell lines.

When Chang liver and M-HeLa cell lines were treated with cisplatin in TSPP–LaSurf compositions in drug doses ranging from one- to one-hundred-fold excess in relation to TSPP, the cytotoxic activity was observed in a dose dependent manner in both cases (Table 2). The cytotoxic effect of cisplatin toward to cancer cells decreases with an increase in its concentration at a constant concentration of TSPP–LaSurf supra-amphiphiles. Such an interesting change in biological properties is probably associated with a change in the ratio of bound/free drug in the system, and the content of triple TSPP–LaSurf–cisplatin complex is optimal at a ten-fold excess of the drug (Table 2). According to the data of spectrophotometric titration of the TSPP:LaSurf = 1:2 complex with cisplatin, it is with a ten-fold excess of the drug that an inflection in the dependence of the absorption intensities with reaching a plateau is observed (Figure 10), and it is likely that one TSPP–LaSurf complex is capable of binding ten molecules of cisplatin. It is nevertheless worth noting the synergism of the therapeutic effect of the triple system TSPP:LaSurf:cisplatin = 1:3:10 in relation to M-HeLa cells, which lasts for two weeks. The IC_50_ value of this freshly prepared system is 4.0 ± 0.20 μM (Table 2), meanwhile, when it is stored in the refrigerator, this value after 1 and 2 weeks is 4.6 ± 0.4 μM and 4.0 ± 0.2 μM, respectively. Maintaining these IC_50_ values for two weeks indicates good drug stability in the TSPP–LaSurf system. Summarizing these data with appropriate analysis and interpretation of spectrophotometric titration and ^1^H NMR spectroscopy (Figure S11), it can be assumed that cisplatin molecules are incorporated into supra-amphiphiles TSPP–LaSurf with the formation of a ternary complex, which improved the therapeutic effect of cancer. Moreover, an increase in the proportion of cisplatin does not significantly affect the IC_50_ value in relation to Chang liver cells, as in the case of cancer M-HeLa cells. An increase in the cytotoxic effect of cisplatin in the presence of supra-amphiphiles TSPP–LaSurf displayed a greater effect towards M-Hela than Chang liver, which indicates a favorable selectivity of the formed drug complexes for diseased cells.

## 4. Conclusions

The construction of supramolecular assemblies composed of the porphyrin and metallosurfactant species has been demonstrated in the bulk aqueous solution for the first time. The results of studies of the interaction of TSPP with LaSurf in an aqueous solution using a set of physicochemical methods showed that at ratios TSPP:LaSurf = 1:1 and 1:2, the supramolecular complexes are formed and can spontaneously assemble into mixed aggregates in an aqueous solution. The LaSurf molecule is able to anchor TSPP molecule around itself by electrostatic and CH–π interactions to form a sandwich-type H-aggregates. An increase in the proportion of LaSurf in a mixture with TSPP leads to the formation of individual LaSurf aggregates, which probably solubilize the TSPP–LaSurf complexes, causing the H-to-J-arrangement transition. Comparing complexes TSPP:LaSurf = 1:1 and 1:2 with each other, one can conclude that the equimolar composition TSPP–LaSurf has greater stability and encapsulating ability towards the model hydrophilic probe rhodamine B. These complexes, in contrast to the pure compounds, are capable of binding the anti-cancer drug cisplatin. Binding of cisplatin by TSPP–LaSurf complexes is accompanied by partial displacement of the LaSurf molecule, but LaSurf is retained in the ternary complex. Considering the targeted effect of porphyrin to cancer cells and the synergistic anticancer effect of cisplatin and lanthanum [48], the revealed physicochemical and biological properties of the ternary systems based on porphyrin, lanthanum-containing surfactant and platinum drug can be useful in creating an effective anticancer therapy.

## Figures and Tables

**Table 1:** Self-diffusion coefficient (D_s_) values for pure TSPP and LaSurf and their mixtures in D_2_O, 300 K.

System	D_s_ (TSPP), 10^−10^ m^2^ s^−1^	D_s_ (LaSurf), 10^−10^ m^2^ s^−1^
Pure solutions	3.35 (0.1 mM)	4.62 (0.3 mM), 1.44 (1 mM)
0.1 mM TSPP–0.3 mM LaSurf	0.82	1.31
0.1 mM TSPP–0.4 mM LaSurf	0.80	1.46
0.1 mM TSPP–1 mM LaSurf	0.80	1.36

**Table 2:** Values of the half-maximal inhibitory concentration IC_50_ (calculated for cisplatin concentration) in Chang liver and M-Hela cells for ternary TSPP−LaSurf–cisplatin systems.

System	IC_50_, μM	
	Chang Liver	M-HeLa
cisplatin [49]	900 ± 71	60 ± 4.8
LaSurf:cisplatin = 3:200 [49]	900 ± 69	60 ± 5.1
TSPP:LaSurf:cisplatin = 1:2:1	2.3 ± 0.11	1.85 ± 0.09
TSPP:LaSurf:cisplatin = 1:3:1	2.6 ± 0.13	2.1 ± 0.11
TSPP:LaSurf:cisplatin = 1:3:10	20.7 ± 1.00	4.0 ± 0.20
TSPP:LaSurf:cisplatin = 1:3:25	12.0 ± 0.60	7.1 ± 0.36
TSPP:LaSurf:cisplatin = 1:3:50	21.1 ± 1.05	15.0 ± 0.75
TSPP:LaSurf:cisplatin = 1:3:100	28.0 ± 1.40	11.0 ± 0.55

## Data Availability

Not applicable.

## Supplementary Materials

The following supporting information can be downloaded at: https://www.mdpi.com/article/10.3390/nano12121986/s1, Figure S1: Concentration dependence of absorbance at 555 nm of rhodamine B water solutions; Figure S2: Plot of (I/I_0_−1)^−1^ versus (C−cmc)^−1^ for the interaction of TSPP and LaSurf; Figure S3: Absorption spectra and corresponding dependence (inset) of the absorption at 404 nm on the concentration of TSPP in a mixture TSPP:LaSurf = 1:2; Figure S4: Particle size distribution for binary TSPP:LaSurf 1:1 (a,b) and 1:2 (c,d) systems (C_TSPP_ = 0.0025 mM) in the absence (a,c) and presence (b,d) of 0.04 mM cisplatin; Figure S5: Dependence of encapsulation efficiency of rhodamine B on TSPP concentration for binary TSPP:LaSurf 1:1 and 1:2 systems; Figure S6: Fluorescence spectra of TSPP in the presence of various amounts of cisplatin (a) and the corresponding dependence of intensity of emission maximum on the concentration of cisplatin (b). C_TSPP_ = 0.01 mM, C_cisplatin_ = 0–0.5 mM; Figure S7: Changes in UV spectra of 0.0025 mM TSPP with increasing cisplatin concentration and corresponding dependence of absorption intensities at 413 nm on cisplatin concentration. The inset shows dependence of absorption intensities at 413 nm in 0.0025 mM TSPP on the cisplatin concentration; Figure S8: (a) Changes in UV spectra of 0.0025 mM TSPP–0.0025 mM LaSurf with increasing cisplatin concentration and (b) corresponding dependence of absorption intensities at 413 nm on cisplatin concentration; Figure S9: Zeta potential for binary TSPP:LaSurf = 1:1 (a,b) and 1:2 (c,d) systems (C_TSPP_ = 0.0025 mM) in the absence (a,c) and presence (b,d) of 0.04 mM cisplatin; Figure S10: Fluorescence spectra of TSPP:LaSurf = 1:1 (a) and 1:2 (b) in the presence of various amounts of cisplatin and the corresponding dependence of intensity of emission maximum on the concentration of cisplatin (c). C_TSPP_ = 0.01 mM, C_cisplatin_ = 0–0.5 mM; Figure S11: ^1^H NMR spectra: (a) 0.1 mM TSPP–0.1 mM LaSurf–0.1 mM cisplatin; (b) 0.1 mM TSPP–0.3 mM LaSurf–0.1 mM cisplatin; (c) 0.1 mM TSPP–1 mM LaSurf–0.1 mM cisplatin in D_2_O; Figure S12: Changes in Q-band region of the absorption spectra of TSPP:LaSurf 1:2 (a) and 1:1 (b) system with increasing cisplatin concentration. The insets show dependences of absorption intensities at 517 nm and 553 nm in 0.0025 mM TSPP–0.005 mM LaSurf on the cisplatin concentration; Figure S13: Fluorescence spectra of TSPP, TSPP:LaSurf = 1:1 and 1:2 (in water (H_2_O) and cell medium (CM)) in the absence and presence of cisplatin (a) and the time dependence of intensity of emission maximum in these solutions (b); Figure S14: Fluorescence spectra of aqueous solutions of TSPP:LaSurf 1:1 (a) and 1:2 (b) diluted two times with water (H_2_O), cell medium (CM), and cell medium without serum (CMwS); Table S1: Hydrodynamic diameter (D_h_) and polydispersity (PdI) index values of aggregates in solutions of pure TSPP and LaSurf, mixed solutions TSPP:LaSurf 1:1 and 1:2 in the absence and presence of cisplatin; Table S2: Self-diffusion coefficients (D_s_) values for TSPP and LaSurf in their mixtures with cisplatin in D_2_O, 300 K; Table S3: Values of the half-maximal inhibitory concentration (IC_50_) in Chang liver and M-Hela cells for free TSPP and binary TSPP−LaSurf systems; Table S4: Values of the half-maximal hemolytic concentration (HC50) in human red blood cells for free TSPP and binary TSPP−LaSurf systems.
