# Supplementary material for: Supramolecular Self-Assembly of Porphyrin and Metallosurfactant as a Drug Nanocontainer Design
Source: Nanomaterials (Basel). 2022 Jun 9;12(12):1986. doi: 10.3390/nano12121986 (PMC9228287; doi:10.3390/nano12121986)
Supplement: Supplementary file 1 [file nanomaterials-12-01986-s001.zip › nanomaterials-1745340-supplementary.pdf]

nanomaterials

MDPI

SUPPORTING INFORMATION

Supramolecular Self-Assembly of Porphyrin and Metallosurfactant as a Drug Nanocontainer Design

Ruslan R. Kashapov [1,*], Yuliya S. Razuvayeva [1], Svetlana S. Lukashenko [1], Syumbelya K. Amerhanova [1], Anna P. Lyubina [1], Alexandra D. Voloshina [1], Victor V. Syakaev [1], Vadim V. Salnikov [2], and Lucia Y. Zakharova [1]

1 Arbuzov Institute of Organic and Physical Chemistry, FRC Kazan Scientific Center of RAS, 8 Arbuzov Street, 420088 Kazan, Russia; razuvayeva.yuliya@iopc.ru (Y.S.R.); lukashenko@iopc.ru (S.S.L.); syumbelya07@mail.ru (S.K.A.); aplyubina@gmail.com (A.P.L.); microbi@iopc.ru (A.D.V.); vsyakaev@iopc.ru (V.V.S.); lucia@iopc.ru (L.Y.Z.)

2 Kazan Institute of Biochemistry and Biophysics, FRC Kazan Scientific Center of RAS, 2/31 Lobachevsky Street, 420111 Kazan, Russia; salnikov_russ@yahoo.com

* Correspondence: kashapov@iopc.ru; Tel.: +7-(843)-273-22-93

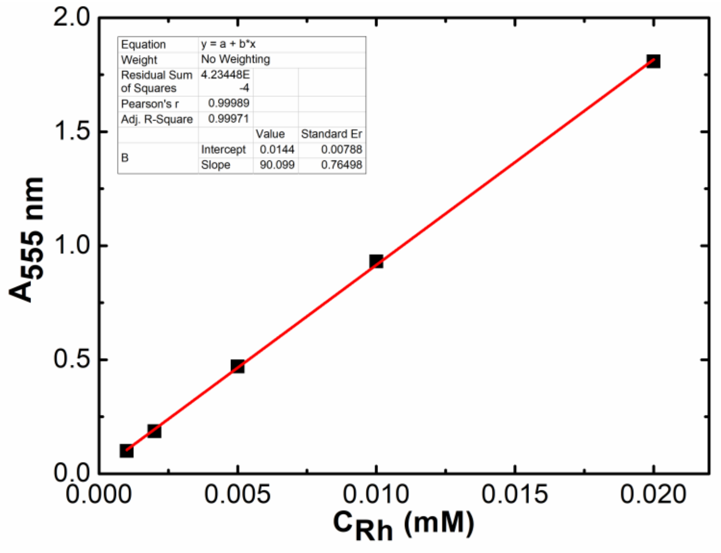

Figure S1. Concentration dependence of absorbance at 555 nm of rhodamine B water solutions.

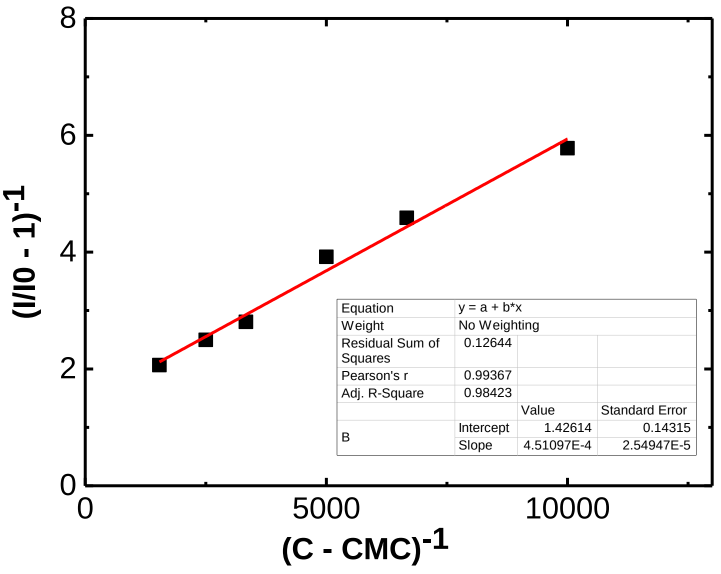

Figure S2. Plot of $(I/I_0{-}1)^{-1}$ versus $(C{-}cmc)^{-1}$ for the interaction of TSPP and LaSurf.

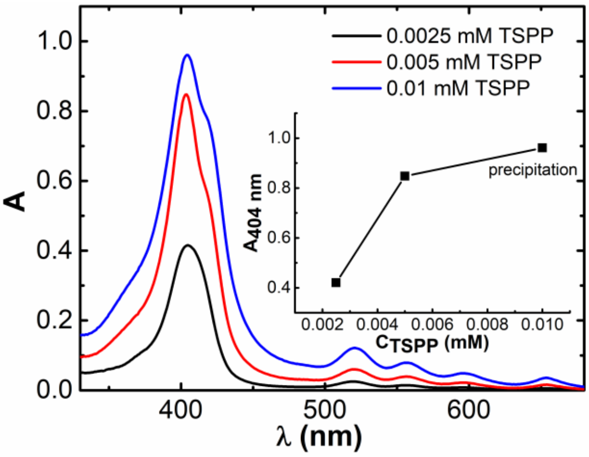

Figure S3. Absorption spectra and corresponding dependence (inset) of the absorption at 404 nm on the concentration of TSPP in a mixture TSPP:LaSurf = 1:2.

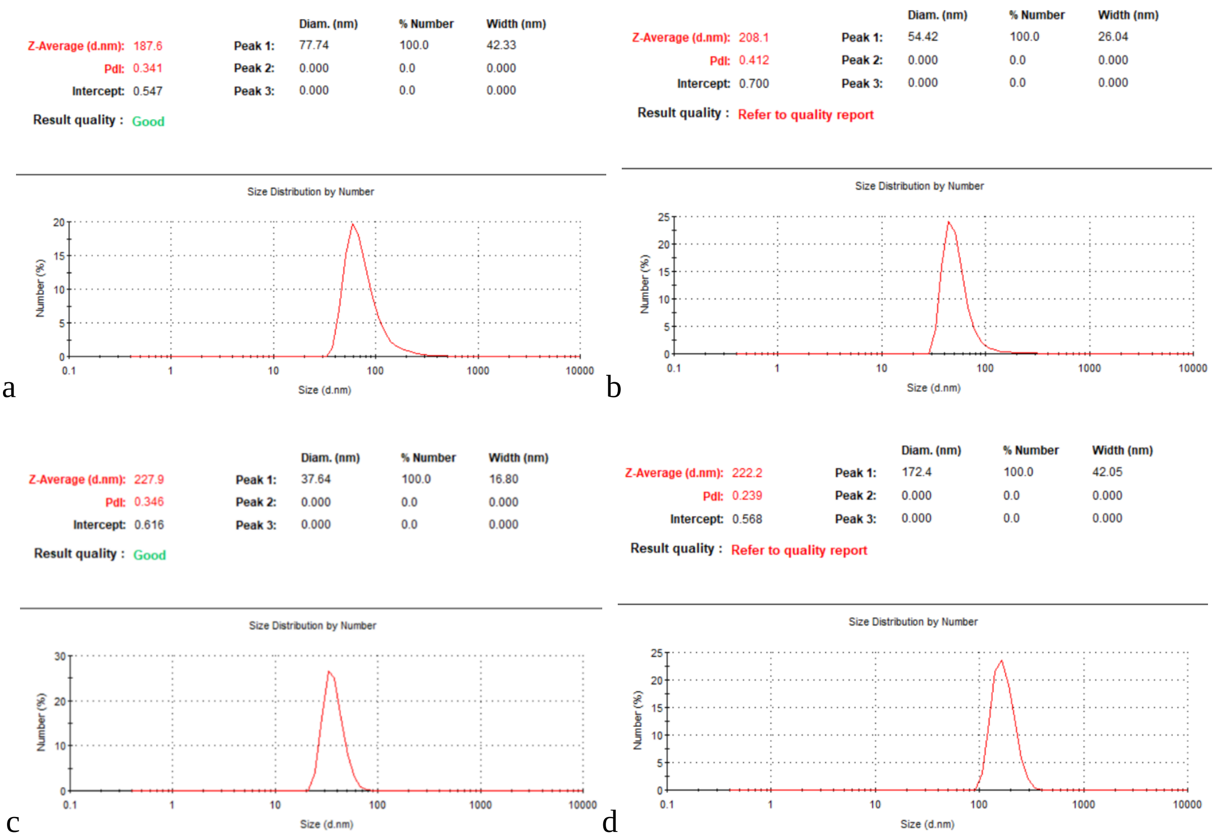

Figure S4. Particle size distribution for binary TSPP:LaSurf 1:1 (**a**, **b**) and 1:2 (**c**, **d**) systems (C_{TSPP} = 0.0025 mM) in the absence (**a**, **c**) and presence (**b**, **d**) of 0.04 mM cisplatin.

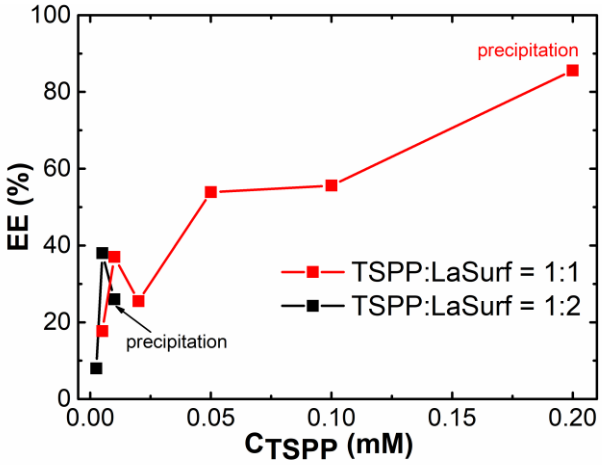

Figure S5. Dependence of encapsulation efficiency of rhodamine B on TSPP concentration for binary TSPP:LaSurf 1:1 and 1:2 systems.

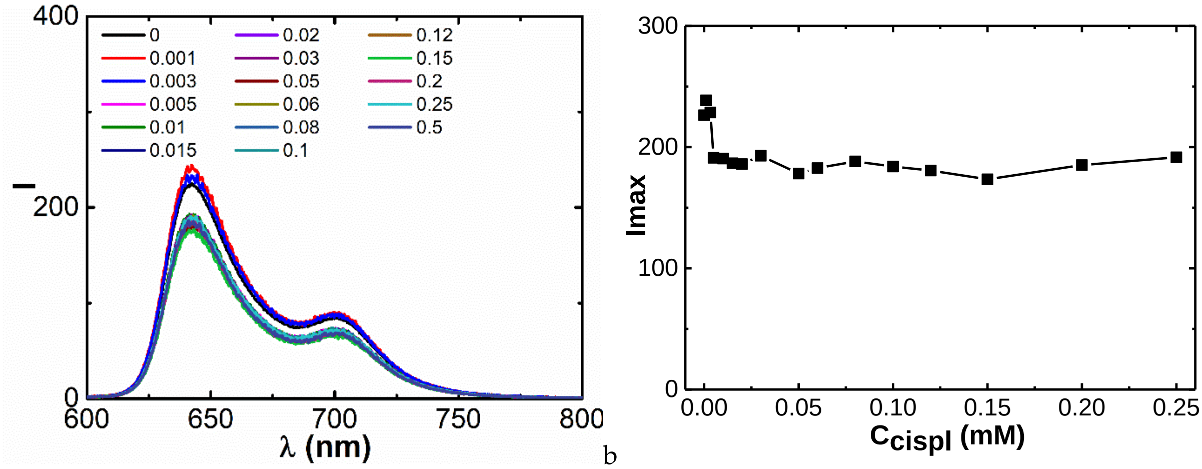

a b

Figure S6. Fluorescence spectra of TSPP in the presence of various amounts of cisplatin (**a**) and the corresponding dependence of intensity of emission maximum on the concentration of cisplatin (**b**). C_{TSPP} = 0.01 mM, $C_{cisplatin}$ = 0–0.5 mM.

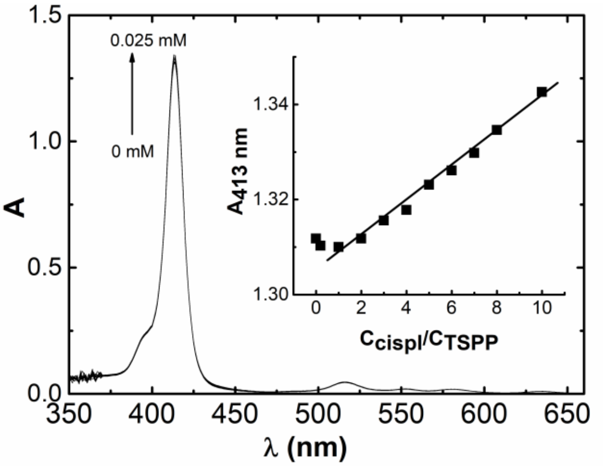

Figure S7. Changes in UV spectra of 0.0025 mM TSPP with increasing cisplatin concentration and corresponding dependence of absorption intensities at 413 nm on cisplatin concentration. The inset shows dependence of absorption intensities at 413 nm in 0.0025 mM TSPP on the cisplatin concentration.

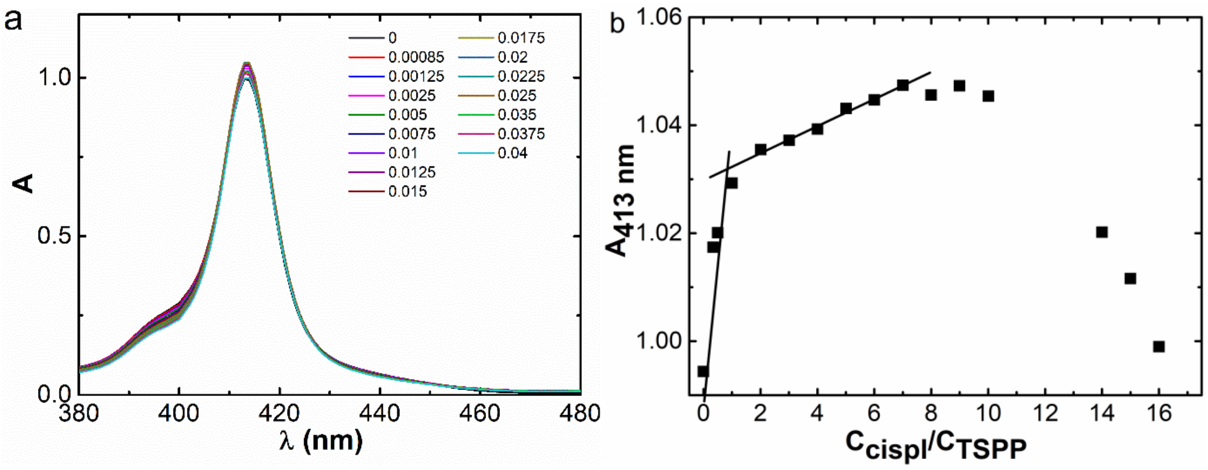

Figure S8. (**a**) Changes in UV spectra of 0.0025 mM TSPP–0.0025 mM LaSurf with increasing cisplatin concentration and (**b**) corresponding dependence of absorption intensities at 413 nm on cisplatin concentration.

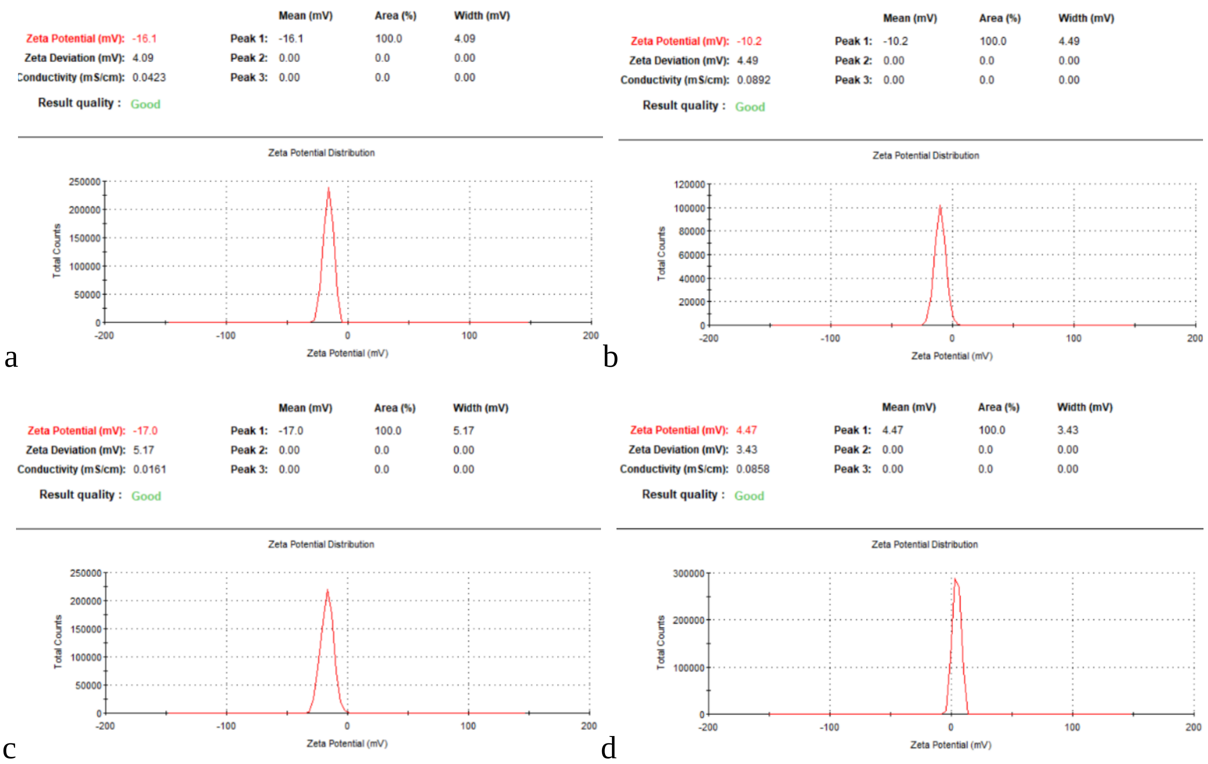

Figure S9. Zeta potential for binary TSPP:LaSurf = 1:1 (**a**, **b**) and 1:2 (**c**, **d**) systems (C_{TSPP} = 0.0025 mM) in the absence (**a**, **c**) and presence (**b**, **d**) of 0.04 mM cisplatin.

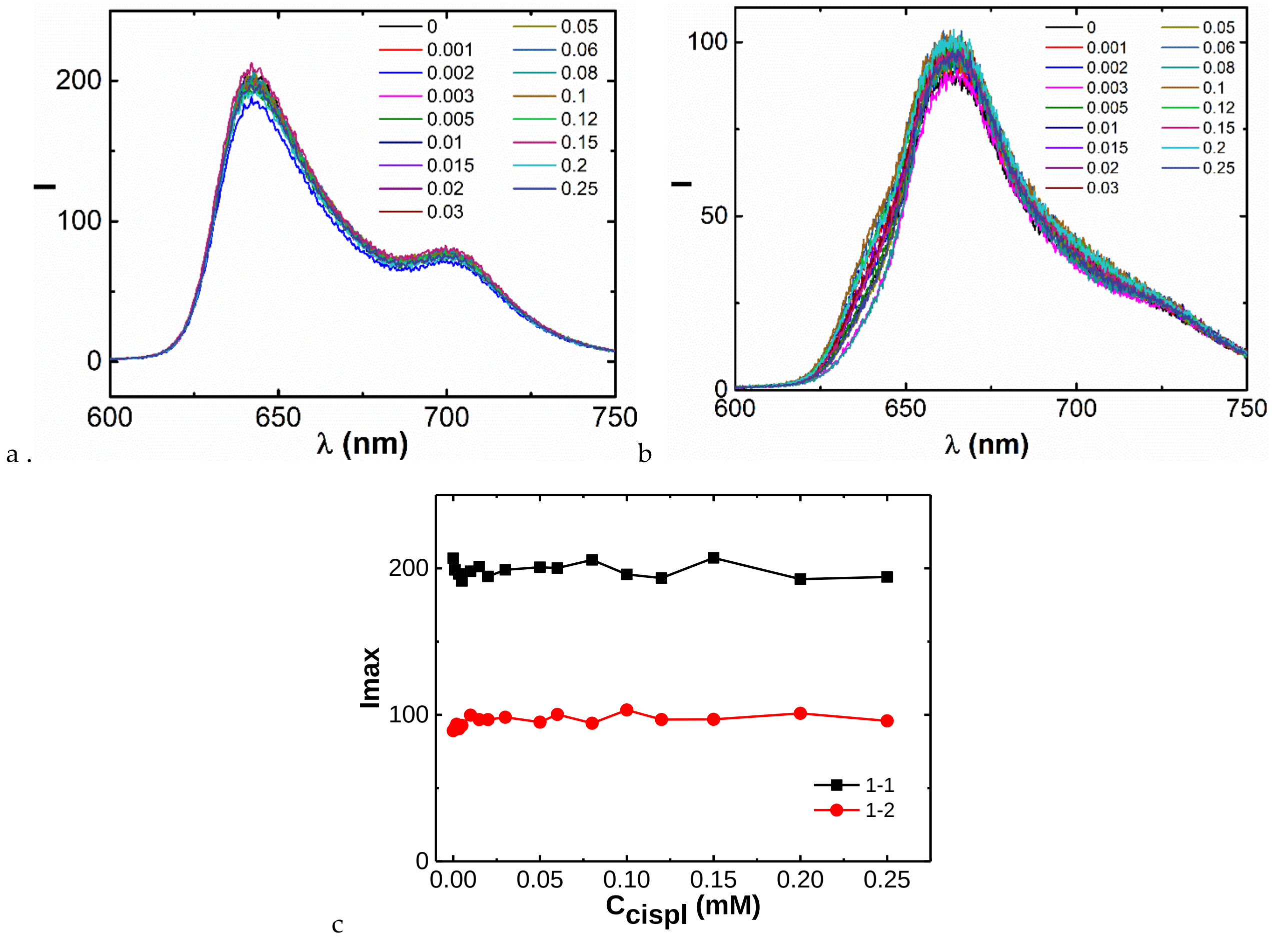

Figure S10. Fluorescence spectra of TSPP:LaSurf = 1:1 (**a**) and 1:2 (**b**) in the presence of various amounts of cisplatin and the corresponding dependence of intensity of emission maximum on the concentration of cisplatin (**c**). C_{TSPP} = 0.0 1 mM, $C_{cisplatin}$ = 0–0.5 mM.

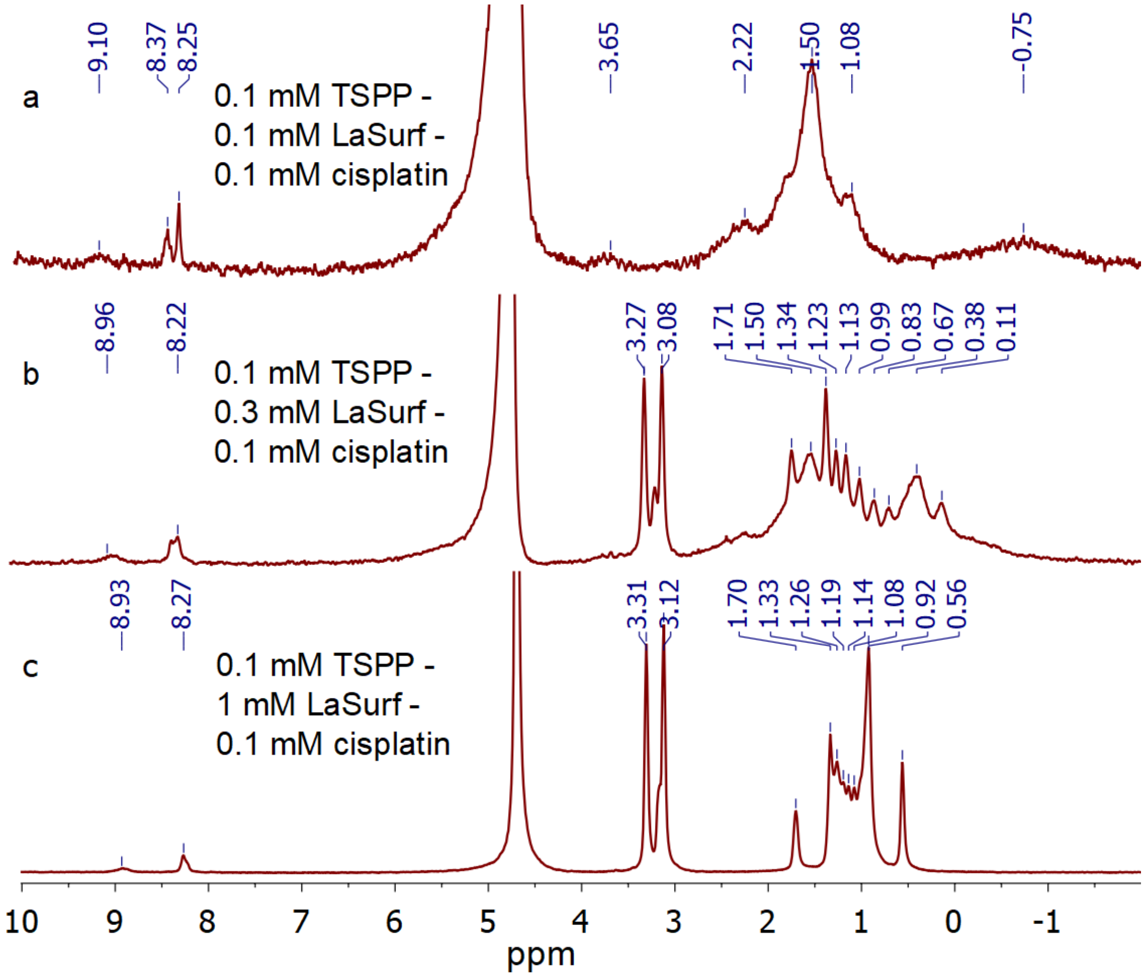

Figure S11. ^{1}H NMR spectra: (**a**) 0.1 mM TSPP–0.1 mM LaSurf–0.1 mM cisplatin; (**b**) 0.1 mM TSPP–0.3 mM LaSurf–0.1 mM cisplatin; (**c**) 0.1 mM TSPP–1 mM LaSurf–0.1 mM cisplatin in D_2O.

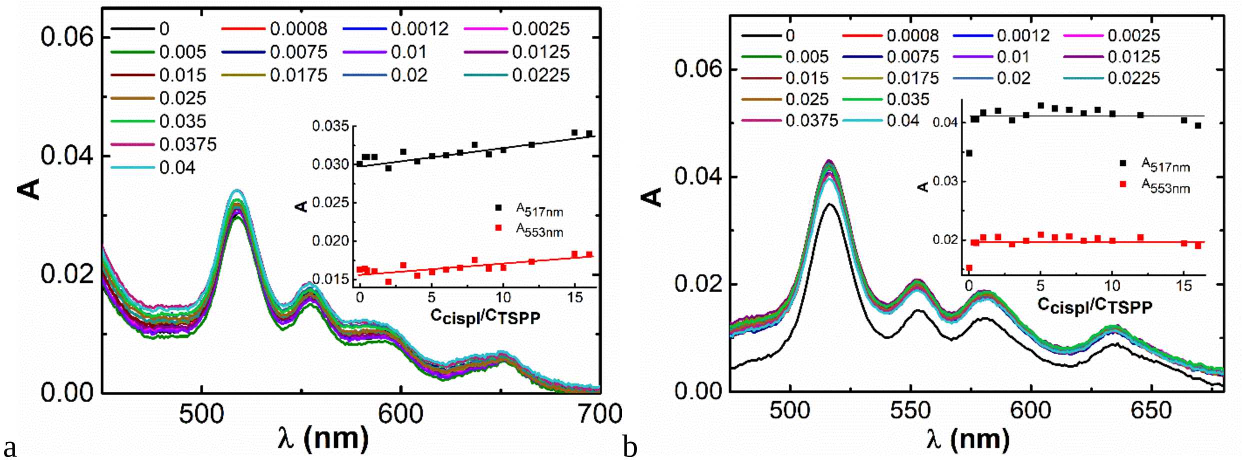

Figure S12. Changes in Q-band region of the absorption spectra of TSPP:LaSurf 1:2 (**a**) and 1:1 (**b**) system with increasing cisplatin concentration. The insets show dependences of absorption intensities at 517 nm and 553 nm in 0.0025 mM TSPP–0.005 mM LaSurf on the cisplatin concentration.

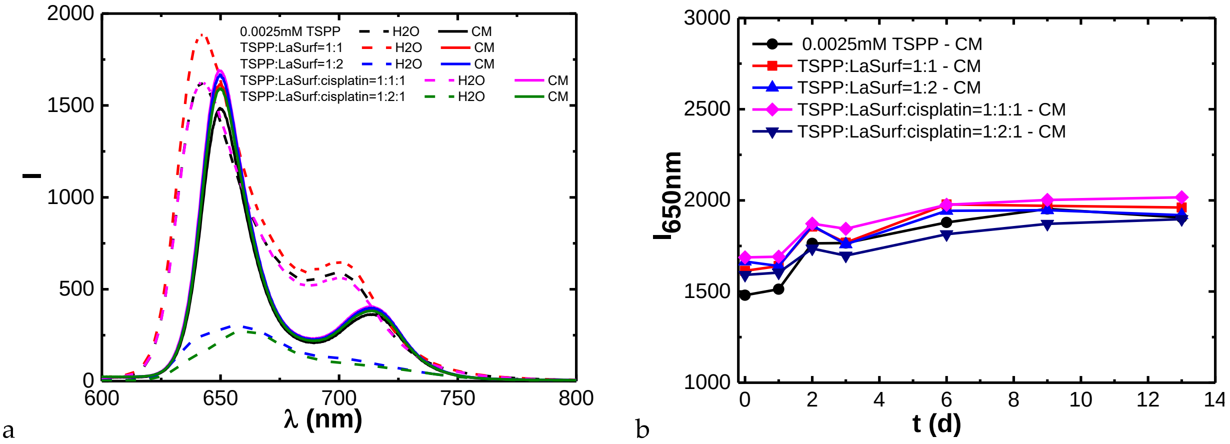

Figure S13. Fluorescence spectra of TSPP, TSPP:LaSurf = 1:1 and 1:2 (in water (H_2O) and cell medium (CM)) in the absence and presence of cisplatin (**a**) and the time dependence of intensity of emission maximum in these solutions (**b**).

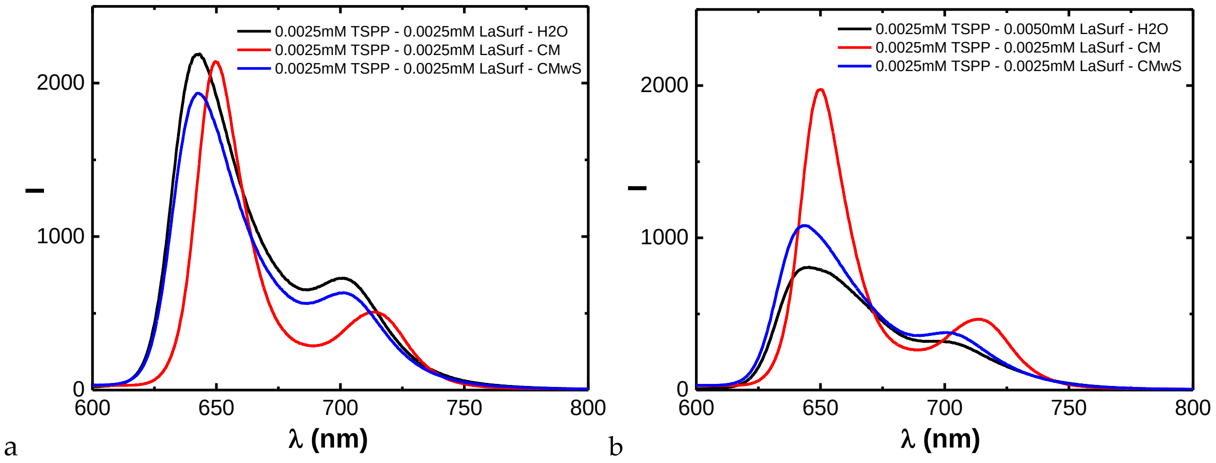

Figure S14. Fluorescence spectra of aqueous solutions of TSPP:LaSurf 1:1 (**a**) and 1:2 (**b**) diluted two times with water (H_2O), cell medium (CM), and cell medium without serum (CMwS).

Table S1. Hydrodynamic diameter (D_h) and polydispersity (PdI) index values of aggregates in solutions of pure TSPP and LaSurf, mixed solutions TSPP:LaSurf 1:1 and 1:2 in the absence and presence of cisplatin.

System	D_h, nm			PdI
	by intensity	by number	by volume	
0.0025 mM TSPP	1.4	1.3	1.3	0.570
0.0025 mM TSPP–0.0025 mM LaSurf	252.4	77.7	362.0 (60%); 93.4 (40%)	0.341
0.0025 mM TSPP–0.0025 mM LaSurf – 0.04 mM cispl	247.7	54.4	324.7 (56%); 67.9 (44%)	0.412
0.0025 mM TSPP–0.005 mM LaSurf	361.6 (90%); 50 (10%)	37.7	42.9 (51%); 431.7 (49%)	0.346
0.0025 mM TSPP–0.005 mM LaSurf – 0.04 mM cispl	196.8	172.4	199.3	0.239

Table S2. Self-diffusion coefficients (D_s) values for TSPP and LaSurf in their mixtures with cisplatin in D_2O, 300K.

	D_s (TSPP), 10^{-10} m^2 s^{-1}	D_s (LaSurf), 10^{-10} m^2 s^{-1}
0.1 mM TSPP–0.3 mM LaSurf–0.1 mM cisplatin	0.81	1.31
0.1 mM TSPP–1 mM LaSurf–0.1 mM cisplatin	0.84	1.23

Table S3. Values of the half-maximal inhibitory concentration (IC_{50}) in Chang liver and M-Hela cells for free TSPP and binary TSPP–LaSurf systems.

System	IC_{50}, μM		HC_{50}, μM
	Chang liver	M-HeLa	erythrocytes
TSPP	>1.25	>1.25	>1.25
TSPP:LaSurf=1:1	>1.25/>1.25	>1.25/>1.25	>1.25
TSPP:LaSurf=1:2	>1.25/>2.5	>1.25/>2.5	>1.25
TSPP:LaSurf=1:3	>1.25/>3.75	>1.25/>3.75	>1.25

Table S4. Values of the half-maximal hemolytic concentration (HC_{50}) in human red blood cells for free TSPP and binary TSPP–LaSurf systems.

System	Concentration, µM		Absorbance at 540 nm	Hemolysis %	HC_{50}, µM
	TSPP	LaSurf			
TSPP	1.25		0.093	4.99	>1.25
	0.625		0.055	0.96	
	0.313		0.047	0	
	0.156		0.047	0	
	0.078		0.047	0	
	0.039		0.047	0	
	0.019		0.047	0	
TSPP:LaSurf=1:1	1.25	1.25	0.103	6.05	>1.25
	0.625	0.625	0.047	0	
	0.313	0.313	0.047	0	
	0.156	0.156	0.047	0	
	0.078	0.078	0.047	0	
	0.039	0.039	0.047	0	
	0.019	0.019	0.047	0	
TSPP:LaSurf=1:2	1.25	2.5	0.083	3.93	>1.25
	0.625	1.25	0.060	1.49	
	0.313	0.625	0.054	0.74	
	0.156	0.313	0.049	0.21	
	0.078	0.156	0.047	0	
	0.039	0.078	0.047	0	
	0.019	0.039	0.047	0	
TSPP:LaSurf=1:3	1.25	3.75	0.070	2.55	>1.25
	0.625	1.875	0.086	4.25	
	0.313	0.9375	0.047	0	
	0.156	0.47	0.047	0	
	0.078	0.234	0.047	0	
	0.039	0.117	0.047	0	
	0.019	0.057	0.047	0	
